# Language and reading in attention‐deficit/hyperactivity disorder and comorbid attention‐deficit/hyperactivity disorder + developmental language disorder

**DOI:** 10.1002/jcv2.12218

**Published:** 2024-04-17

**Authors:** Kaitlyn M. A. Parks, Janis Oram Cardy, Marc F. Joanisse

**Affiliations:** ^1^ Department of Psychology Western University London Ontario Canada; ^2^ Brain and Mind Institute Western University London Ontario Canada; ^3^ School of Communication Sciences and Disorders Western University London Ontario Canada

**Keywords:** attention‐deficit/hyperactivity disorder, developmental language disorder, language, sight word reading, specific language impairment

## Abstract

**Background:**

The current study sought to examine whether psycholinguistic assessments could discriminate children and adolescents with developmental language disorder (DLD) from those with attention‐deficit/hyperactivity disorder (ADHD; combined or inattentive subtype) and comorbid DLD + ADHD.

**Methods:**

The Clinical Evaluation of Language Fundamentals—Screening Test (CELFST; Wiig et al., 2013), the Comprehensive Test of Phonological Processing (*nonword repetition* subtest; Wagner et al., 2013), and the Test of Word Reading Efficiency (*sight word* and *phonemic decoding* subtests; Torgesen et al., 2012) were examined in 441 children and adolescents between 6 and 16 years of age.

**Results:**

The presence of a language disorder (with or without ADHD) predicted poor performance across tasks. Children and adolescents with ADHD (combined vs. inattentive) only significantly differed in sight word reading, in favor of those with combined type. Measures of reading efficiency could distinguish between the two types of ADHD, but not between other groups. Interestingly, scores on the standard language screener were no worse for children with ADHD + DLD than children with DLD only.

**Conclusions:**

The combination of comorbid ADHD + DLD did not appear to be associated with lower language abilities, sight word reading, or phonemic decoding relative to DLD alone. Reading efficiency was effective in discriminating between ADHD subtypes. These findings offer valuable insights into differential diagnosis and the identification of comorbidity.

**Key points**

- Poor language, nonword repetition, and reading efficiency have been reliably associated with language impairment in children and adolescents.
- The diagnostic utility of psycholinguistic assessments to differentiate children with developmental language disorder (DLD) from other common childhood disorders such as attention‐deficit/hyperactivity disorder (ADHD) is promising but far too limited.
- Going forward, the inclusion of children with comorbidities and unique subtypes must be considered for research findings to be generalizable to broader DLD and ADHD populations.
- Key messages to researchers and practitioners include awareness of which tests are and are not useful for differential diagnosis to ensure that each child receives services that are tailored to their specific needs.

## INTRODUCTION

One key issue for researchers when administering diagnostic assessments is not whether a child has typical or atypical development, but which designation the child falls under. Making these distinctions can be especially difficult in children with attention‐deficit/hyperactivity disorder (ADHD) and developmental language disorder (DLD) because elevated levels of inattention in ADHD could disrupt the way these children respond on language assessments in a way that is like DLD. The significant social and academic difficulties resulting from poor language skills have led researchers to investigate which measures are most effective at identifying language impairments (Conti‐Ramsden, 2003; Poll et al., 2010; Redmond et al., 2011). Previous research has predominately focused on DLD, with some comparing the linguistic characteristics of ADHD and DLD (Parigger et al., 2012; Redmond et al., 2011, 2019; Stanford & Delage, 2020, 2023). Given that language deficits are prevalent in both disorders, it is unsurprising that assessments of language function can serve as reliable diagnostic markers. Children with DLD exhibit clear deficits in areas such as verbal comprehension (Bishop et al., 2017; Korkman & Hakkinenrihu, 1994), grammar (Fonteneau & van der Lely, 2008; Hsu & Bishop, 2010), and the ability to make accurate vocabulary associations (see Leonard, 2014 for a review).

While children with ADHD also struggle in these language domains, their difficulties are often less pronounced than those seen in children with DLD (Oram Cardy et al., 2010). This distinction in language profiles makes it easier to differentiate between the two groups. In addition to comprehensive language assessments, research spanning several decades has shown that nonword and sentence repetition tasks can be particularly valuable indicators of language impairment. Several key studies have found associations between performance on these tasks and genotype/phenotype correspondence in DLD (Falcaro et al., 2008; Monaco, 2007; Redmond et al., 2011; Rice et al., 2009). Nonword and sentence repetition tasks have consistently proven to be reliable clinical markers of language impairment (Archibald & Joanisse, 2009) across various age groups, including young adults (Poll et al., 2010) and young children (Conti‐Ramsden, 2003; Conti‐Ramsden & Hesketh, 2003; Oetting & Cleveland, 2006; Redmond et al., 2011). Moreover, these tasks have demonstrated their effectiveness in different language contexts, including English (Redmond et al., 2011), Danish (Vang Christensen, 2019), Arabic (Taha et al., 2021), Italian (Guasti et al., 2021), and Mandarin‐speaking children (Wang et al., 2022).

Word and nonword reading abilities might also serve as important markers of DLD. However, compared to nonword repetition and general language skills, less research has focused on using reading measures as clinical markers for DLD. Most studies examining reading efficiency have primarily investigated children and young adults with dyslexia, a disorder characterized by marked difficulties in decoding, word reading, and spelling (APA, 2013). Dyslexia is estimated to affect between 3% and 20% of the population (Rutter et al., 2004; Shaywitz, 1996; Spencer et al., 2014), although prevalence rates can vary depending on diagnostic criteria.

Interestingly, both children with ADHD (Brimo et al., 2021; Gillberg, 2010; Kaplan et al., 2001; Peterson & Pennington, 2015) and DLD (Catts et al., 2005) also frequently experience co‐occurring reading difficulties. Comorbidity rates are significant, with 25%–40% of children with ADHD (Boada et al., 2012) and 50% of those with DLD presenting with reading difficulties (Adlof & Hogan, 2018). Notably, research has indicated that reading problems in individuals with ADHD are more associated with the inattention components of the disorder rather than the hyperactive‐impulsive aspects (Germanò, et al., 2010; Mascheretti et al., 2017). Previous work has further shown that teacher ratings of behavioral inattention have a direct effect on sight word reading (Martinussen et al., 2014). Further research is needed to better understand how the specific components of ADHD can differentially impact reading outcomes. Investigating the unique reading challenges faced by children with primarily inattentive symptoms in ADHD compared to other subtypes will enhance our understanding of how these specific subtypes impact reading outcomes. This research will contribute to the development of targeted interventions for addressing reading difficulties in children with ADHD, tailoring approaches based on the needs associated with different ADHD subtypes.

Failing to consider how assessments can differentiate between comorbid samples and pure samples limits the generalizability of findings to the larger population, where comorbidity rates are high. There is a significant overlap in symptomology between children with ADHD and DLD, underscoring the need to identify the best methods for distinguishing between these conditions. Since most assessments are not designed to distinguish clinical groups (differential diagnosis), it can leave the source of poor performance open to the examiner's interpretation, potentially leading to misdiagnosis. Accurate diagnosis is crucial in ADHD and DLD, as children often face under‐identification (McGregor, 2020) or excessive referrals for evaluations (Gascon et al., 2022). The outcome of diagnostic assessments can be life‐altering; they impact the kind of services children will and will not receive. The standard interventions associated with the two conditions are vastly different, thus it is clinically important to identify measures capable of distinguishing cases of ADHD from cases of DLD. There may be substantial overlap in their symptomology, but the sources of these problems are likely different and require unique responses. Therefore, exploring the effectiveness of specific assessments in differentiating between ADHD and DLD can inform future protocols, leading to improved treatments and services for these children.

The objectives of this study were to first investigate if the presence of a comorbid language disorder in individuals with ADHD exacerbates language and reading difficulties and second, determine whether psycholinguistic measures can aid in distinguishing between ADHD and/or DLD. To achieve these goals, we analyzed data from a large‐scale open dataset to assess the discriminative abilities of language assessments, nonword repetition, Phonemic Decoding Efficiency (PDE), and Sight word Efficiency (SWE). We focused on distinguishing cases of DLD, ADHD (combined and inattentive subtypes), and comorbid ADHD + DLD in children and adolescents aged 6–16 years. Additionally, we aimed to identify the most effective combination of these measures for accurately identifying cases of DLD.

## METHOD

### Study sample

All participants were enrolled in the Healthy Brain Network (HBN) database (Alexander et al., 2017). The HBN, launched by The Child and Mind Institute, is an open database that contains data from approximately 10,000 New York area children and young adults across various measures. Participants were recruited using a community referral recruitment model. Advertisements were distributed to families who had concerns about psychiatric symptoms in their child. Participation was therefore based on perceived clinical concern, resulting in a high proportion of individuals affected by psychiatric illness in the HBN sample. Exclusion criteria included serious neurological disorders, moderate to severe cognitive impairment (i.e., IQ below 66), and uncorrected hearing or visual impairment (Alexander et al., 2017). Participants taking stimulant medication were instructed to suspend use during testing.

All HBN participants received a base battery of assessments that took approximately 12 h to complete. For a subset of conditions, including language disorder, those whose performance on the base battery suggested possible impairments completed additional clinical diagnostic assessments for that domain of concern (Alexander et al., 2017). Results on the assessments were used by a clinical team to make consensus diagnoses where appropriate, which were then recorded in the HBN database. Although participants in the HBN dataset completed a battery of tests, only those relevant to the current study are described here. See Alexander et al. (2017) for further details on recruitment, eligibility, and diagnostic procedures.

Participants selected from the HBN database for the current study were children and adolescents between 6 and 16 years of age (*M* = 9.73 years, *SD* = 2.56; 75.7% male) who had a confirmed diagnosis of ADHD combined subtype (ADHD‐C), ADHD inattentive subtype (ADHD‐I), and/or language disorder. The HBN dataset also included participants with confirmed diagnoses of ADHD hyperactive‐impulsive subtype (*n* = 17); however, these participants did not have complete data on the tasks needed for the current study and were therefore excluded. We also excluded participants with additional diagnoses of intellectual disorder, autism spectrum disorder, selective mutism, schizophrenia, and other psychotic disorders. Other co‐occurring diagnoses (e.g., specific learning disorder, speech sound disorder, anxiety disorder) were not excluded. Participants who did not have complete demographic information (*n* = 179) and those who were diagnosed with “other specified ADHD” or “other specified ADHD + language disorder” were also excluded. Given that our included participants with language disorder had no hearing impairment, neurological disorder, intellectual impairment, or autism, they met criteria for DLD according to its international consensus definition (Bishop et al., 2017). Our final sample included participants with ADHD‐C (*n* = 148), ADHD‐I (*n* = 192), DLD (*n* = 39), ADHD‐C + DLD (*n* = 28), and ADHD‐I + DLD (*n* = 34). See Table 1 for demographic information for each diagnostic category.

**TABLE 1:** Demographic information for each diagnostic group (*N* = 441).

Diagnosis	*n*	Mean age *(SD)*	Min‐max
ADHD‐C	148	9.21 (2.29)	6.00–15.30
ADHD‐I	192	10.40 (2.61)	6.01–16.00
DLD	39	8.51 (2.34)	6.03–15.70
ADHD‐C + DLD	28	8.91 (2.43)	6.01–14.10
ADHD‐I + DLD	34	10.1 (2.66)	6.32–15.90

*Note*: Age is in years.

Abbreviations: ADHD‐C, Attention‐deficit/hyperactivity disorder combined subtype; ADHD‐I, Attention‐deficit/hyperactivity disorder inattentive subtype; DLD, Developmental language disorder.

### Reference standard for ADHD and DLD status

To determine ADHD status, clinicians and trained research assistants administered the following tasks to all participants: Conners ADHD Rating Scales Self‐Report (Conners), Strengths and Weaknesses of ADHD Symptoms and Normal Behavior Scale, Quotient ADHD System, and the Schedule for Affective Disorders and Schizophrenia‐Children's version (KSADS‐COMP). A clinical team evaluated all data to arrive at a consensus, or to rule out, diagnosis of ADHD‐C or ADHD‐I.

Determination of language disorder status was a two‐part process. First, all participants completed the Clinical Evaluation of Language Fundamentals, 5th edition (CELF‐5) Screening Test (CELFST; Semel et al., 2013) and the Goldman Fristoe Test of Articulation, 3^rd^ edition (GFTA‐3; Goldman, 2015) *Sounds and Words* subtest. Those who failed the Clinical Evaluation of Language Fundamentals Screening Test (CELFST) and/or performed poorly on the GFTA‐3 completed an extended evaluation with a licensed speech‐language pathologist that included the full CELF‐5 (Wiig et al., 2003), Expressive Vocabulary Test (EVT; Williams, 2007), the Peabody Picture Vocabulary Test (PPVT; Dunn & Dunn, 2007), the CELF‐5 Metalinguistics (Wiig et al., 2003), and additional subtests of the GFTA‐3. Performances on these measures were used to assign a clinical consensus diagnosis of language disorder (and/or speech sound disorder). Unlike the determination of ADHD status, the evaluation measures used to diagnose language disorder was not used to rule‐out language disorder. Instead, those who passed the CELFST and performed within age expectations on the GFTA‐3 were considered to not have a speech or language disorder, and no further assessments were completed. Therefore, the reference standard for DLD diagnosis included performance on the extended language evaluation battery and clinical judgment whereas ruling‐out of DLD diagnosis used performances on the CELFST and GFTA‐3.

Although the sample sizes for our comorbid groups were smaller (ADHD‐C + DLD *n* = 28; ADHD‐I + DLD *n* = 34) than the remaining groups, it was important to maintain them as distinct diagnostic categories in line with the HBN evaluations. The primary focus of this paper was to distinguish between ADHD and comorbid ADHD + DLD, however, comparisons were also made of the two subtypes of ADHD to investigate how symptoms of each subtype may relate to language and reading outcomes.

### Index measures

***Cognitive measures.*** All participants completed The *Wechsler Intelligence Scale for Children* as a test of general intelligence (WISC‐V; Wechsler, 2014), which provides norms from 6.0 to 17.0 years of age. We elected to use the *Visual Spatial Index* (VSI) score rather than Full Scale IQ score in our analyses. The VSI provides an estimate of children's nonverbal reasoning and concept formation skills. It evaluates visual perception and organization, visual motor coordination, and the ability to synthesize abstract visual concepts and information. Since this index generates an IQ score that does not rely on verbal responding, it provides an estimate of IQ that is not confounded by language difficulties, which is of particular importance for the groups with DLD (DeThorne & Schaefer, 2004).

***Psycholinguistic measures.*** The *CELFST* (Semel et al., 2013) provides a brief but comprehensive assessment of language abilities and helps to evaluate whether a child needs further testing to identify a potential language disorder. The assessment includes the most discriminating items from the full CELF‐5 assessment and evaluates knowledge of grammatical morphemes, vocabulary associations, interpretation of spoken directions, and verbal sentence repetition. As a criterion‐referenced test, it generates a total raw score, which is then evaluated against an age‐based cutoff, that is, to determine pass/fail of the screening. Note that there is partial overlap between the references measures for DLD status and this index measure in that all participants in the ADHD‐C and ADHD‐I groups would, by definition, have received a raw score above the CELFST cutoff. By contrast, it was not necessary for those in the DLD, ADHD‐C + DLD, and ADHD‐I + DLD groups to fail the CELFST to proceed to full evaluation and subsequently receive a clinical diagnosis of language disorder (poor performance on the GFTA‐3 alone would have prompted the full evaluation). Nonetheless, it is reasonable to expect that the groups with DLD would have lower scores on the CELFST than the two groups without DLD in this study. However, comparisons within these groups (i.e., between groups with ADHD‐C vs. ADHD‐I and between DLD vs. ADHD‐C + DLD and ADHD‐I + DLD) on the CELFTST are of key interest.

The *Nonword Repetition* subtest of the *Comprehensive Test of Phonological Processing*, Second Edition (CTOPP‐2; Wagner et al., 2013) was used to evaluate children's phonological memory abilities as a prerequisite to reading fluency. The nonword repetition subtest measures the ability to repeat nonwords of increasing length and was included here given its sensitivity to both reading and language difficulties in children.

The *Test of Word Reading Efficiency*, Second Edition (TOWRE‐2; Torgesen et al., 2012) provides an assessment of single‐word and nonword reading fluency. The *SWE* and *PDE* subtests were included in analyses and evaluated the number of real words (SWE) and nonwords (PDE) an individual can correctly name in 45 s.

## ANALYSES

### Comparing language and reading performance

***Analysis of variance*.** Univariate analysis of variance (ANOVA) was conducted to determine the presence of group differences on the psycholinguistic measures (CELFST, NWR, SWE, and PDE). For CELFST, Levene's test indicated homogeneity of variance was violated, and therefore Welch's robust test of equality of means (asymptotically F distributed) was performed to determine group differences, and follow‐up Games‐Howell analysis was used to identify pairwise comparisons.

To evaluate the minimum sample size required to test these comparisons, an a priori power analysis was performed using G*Power version 3.1.9.7 (Faul et al., 2007). According to Cohen's (1988) guidelines, the study determined that a total sample of 200 participants (with approximately 40 participants per group) was needed to achieve 80% power to detect a medium effect size. The significance criterion for the ANOVA was set at *α* = .05. The obtained sample size for the study was *N* = 441, which was considered adequate for testing the comparisons. However, the comorbid groups (*n* ADHD‐I + DLD = 34; *n* ADHD‐C + DLD = 28) had smaller sample sizes compared to the other groups. A power calculation determined that the minimum effect size detectable with the smallest group (*n* = 28) would need to be at least medium‐large sizes (*d* = 0.61). While comparisons involving these samples could yield valuable insights, it is important to consider the limitations imposed by our sample size when interpreting the results, especially for practical applications. Specifically, small or medium effects were not detectable here.

### Diagnostic utility

***Receiver operating curves.*** To examine how well the assessments could accurately distinguish diagnostic cases, receiver operating curves (ROC) were generated. The diagnostic power and optimal clinical cut‐off values for each specific test are reported (Perkins & Schisterman, 2006; Sackett et al., 1991) for the following discriminations: ADHD‐C versus ADHD‐C + DLD, ADHD‐C versus DLD, ADHD‐I versus DLD, ADHD‐C + DLD versus DLD, ADHD‐C + DLD versus ADHD‐I, ADHD‐C versus ADHD‐I, ADHD‐C versus ADHD‐I + DLD, ADHD‐I versus ADHD‐I + DLD, and ADHD‐I + DLD versus DLD (Please see Figures S6–S14 for ROC curves). Given that sensitivity and specificity are valued equally in the current study, the index of union (IU) method (Unal, 2017) was used for selecting optimal cut‐off points for diagnostic tests (See Table 3 for optimal cut‐off points, sensitivity, specificity, positive likelihood ratio, and negative likelihood ratio). Following Simundic (2012), we interpreted area under the curve values between 0.9 and 1.0 as indicating excellent diagnostic accuracy, 0.8–0.9 as very good, between 0.7 and 0.8 as good, 0.6–0.7 as sufficient, 0.5–0.6 as bad, and <0.5 not useful.

An additional power analysis was performed using MedCalc Statistical Software version 19.2.6 (MedCalc Software by Ostend, Belgium; https://www.medcalc.org; 2020) to evaluate the minimum sample size needed for the ROC analysis with a Type I error set at *α* = .05 and Type II error set at *β* = .80. The analysis indicated that a minimum of 20 negative cases (no diagnosis) and 10 positive cases were needed to achieve these (total *N* = 30).

### Predicting DLD status

***Binary logistic regression.*** A binary logistic regression was performed to determine whether several cognitive assessments could predict DLD status. The following variables were entered as predictors: WISC VSI (nonverbal IQ), CELFST, SWE, PDE. Specifically, we aimed to compare cases of ADHD without DLD (combined and inattentive; *n* = 340) with cases of DLD (with or without ADHD; *n* = 101) to determine whether a specific combination of assessments (WISC VSI, CELFST, SWE, PDE, NWR) could better predict DLD status compared to individual assessments alone. Collinearity, which indicates multicollinearity in regression analysis, was tested, and all variables had variance inflation factors below 5.00, indicating low collinearity between predictors (James et al., 2017). To estimate the internal validity of the regression analysis findings, a bias‐corrected accelerated (BCa) bootstrap method was applied. The BCa approach was selected because it provides more accurate estimation of 95% confidence intervals compared to the percentile approach (Efron, 1987; Efron & Tibshirani, 1993; Jung et al., 2019).

Cross validation methods help to estimate error bias, determine the best model fit, and ensure the model is not overfitting the data. To evaluate how well our logistic model would perform in practice and make more precise conclusions about the model predictions, we applied a leave‐one‐out (LOOCV) cross validation method to our data. In terms of evaluation metrics, the root mean square error (RMSE) is the most acceptable and measures the average difference between the predictions made by the model and the real observations. RMSE values between 0.2 and 0.5 indicate that the model can predict the data accurately.

For logistic regressions, sample size recommendations are less clear because odds ratios are scale dependent and there is no well‐defined *R* ^2^. Thus, the minimum required sample size for our analysis was determined using rule‐of‐thumb guidelines (Bujang et al., 2018; Peduzzi et al., 1996) using an event per variable formula. The ideal sample size is calculated using the following formula: (*n* = 100 + 50*i*) where *i* is equal to the number of predictors in the final model. For the current analysis, the EVP value is equal to 350 and our total sample size is 441 (ADHD *n* = 340; DLD *n* = 101). These findings suggest that our sample size is sufficient to perform our regression analysis.

## RESULTS

### Comparing performance across cognitive and psycholinguistic assessments

Group means and performance summaries for each cognitive and psycholinguistic assessment are presented in Table 2. There were significant group differences in nonverbal IQ, *F* (4, 397) = 7.09, *p* < .001. Pairwise comparisons revealed significant differences between ADHD‐C and ADHD‐C + DLD (*p* < .05), ADHD‐C and DLD (*p* < .001), ADHD‐I and DLD (*p* = <.05), as well as between ADHD‐C and ADHD‐I + DLD (*p* < .01). No other significant group differences were observed (Please see Figures S1–S5 for plots illustrating the distribution of all assessments).

**TABLE 2:** Performance across all assessments by group.

Variable	ADHD‐C (*n* = 147)	ADHD‐I (*n* = 192)	ADHD‐C + DLD (*n* = 28)	ADHD‐I + DLD (*n* = 34)	DLD (*n* = 39)	*df*	*F*
Nonverbal IQ^a^	103 (15.70)	92.70 (18.60)	100.00 (14.40)	92.60 (14.50)	91.80 (13.60)	4, 397	7.09***
Language^b^	16.90 (4.96)	12.0 (4.17)	17.70 (5.54)	11.30 (3.62)	10.70 (3.16)	4, 85.90	45.49***
Nonword repetition^b^	7.74 (3.09)	6.05 (3.91)	7.55 (2.93)	5.61 (2.76)	5.47 (3.23)	4, 391	7.28**
Phonemic decoding^a^	102.00 (16.10)	83.90 (13.80)	99.30 (13.50)	91.90 (18.00)	91.40 (15.40)	4, 375	8.89***
Sight word reading^a^	108.00 (16.30)	89.60 (16.60)	102.00 (15.10)	95.80 (16.50)	85.90 (15.60)	4, 377	10.70***

*Note*: Means presented and standard deviations in brackets.

Abbreviations: Language, Clinical Evaluation of Language Fundamentals Screening Test; Nonverbal IQ, Wechsler Intelligence Scale Visual Spatial Index; NWR, Comprehensive Test of Phonological Processing Nonword repetition subtest; Phonemic Decoding = Test of Word Reading Efficiency Phonemic decoding subtest; Sight Word Reading = Test of Word Reading Efficiency Sight word subtest.

^a^ Standard Score with *M* = 100, *SD* = 10.

^b^ Scaled Score with *M* = 10, *SD* = 3.

*<.05, **<.01, ***<.001.

Language scores showed significant differences among groups, *F* (4, 85.90) = 45.49, *p* < .001. Pairwise comparisons indicated the ADHD‐C group had higher scores than the groups with DLD (*p* < .001), ADHD‐C + DLD (*p* < .001), and ADHD‐I + DLD (*p* < .001). Similarly, the group with ADHD‐I performed better than those with DLD (*p* < .001), ADHD‐C + DLD (*p* < .001), and ADHD‐I + DLD (*p* < .001). Of note, the groups with ADHD‐C and ADHD‐I did not differ on the CELFST, nor did the groups with DLD, ADHD‐C + DLD, and ADHD‐I + DLD.

In the nonword repetition task, significant group differences were observed *F* (4, 391) = 7.28, *p* = <.002. Pairwise comparisons further revealed significant differences between ADHD‐C versus DLD (*p* = <.001), ADHD‐C versus ADHD‐I + DLD (*p* = .005), ADHD‐I versus DLD (*p* = .002), and ADHD‐I versus ADHD‐I + DLD (*p* = .010).

In terms of SWE, significant group differences were observed, *F* (4, 377) = 10.70, *p* = <.001. Pairwise comparisons further indicated there were significant differences between ADHD‐C and DLD (*p* = .002), ADHD‐C and ADHD‐C + DLD (*p* < .001), ADHD‐C and ADHD‐I (*p* = .011), and ADHD‐C and ADHD‐I + DLD (*p* < .001). Additionally, significant differences were found between ADHD‐C + DLD and ADHD‐I (*p* = .019), as well as between ADHD‐I and ADHD‐I + DLD (*p* = .023).

In the analysis of PDE, significant differences were found between groups, *F* (4, 375) = 8.89, *p* = <.001. Further pairwise comparisons revealed that individuals with ADHD‐C significantly differed from those with DLD (*p* = .005), ADHD‐C + DLD (*p* < .001), and ADHD‐I + DLD (*p* = .001). There were also significant differences between ADHD‐C + DLD and ADHD‐I (*p* < .001).

In summary, children with ADHD‐C and ADHD‐I demonstrated better performance across various language and reading domains compared to other diagnostic groups. However, there were no significant differences between ADHD‐C and ADHD‐I, except for the SWE task, where ADHD‐C performed better. Both children with ADHD‐C and ADHD‐I showed better performance on the nonword repetition task compared to individuals with DLD and ADHD‐I + DLD. However, children with comorbid ADHD‐C + DLD did not perform worse than those with ADHD‐C or ADHD‐I. On the SWE task, children with ADHD‐C had superior abilities compared to the other diagnostic groups, including ADHD‐I. However, children with ADHD‐I still outperformed the comorbid groups of ADHD‐C + DLD and ADHD‐I + DLD. Individuals with ADHD‐C showed better phonemic skills compared to other diagnostic groups, except for ADHD‐I, and children with ADHD‐I outperformed the ADHD‐I + DLD on this measure.

The collective results indicate that individuals with DLD (with or without ADHD) exhibited the most significant difficulties across the assessed measures. Those with DLD or comorbid ADHD + DLD (both combined and inattentive subtypes) had the lowest scores across tasks. The presence of comorbid ADHD + DLD did contribute to poorer scores on assessments, but this effect was not more pronounced than in cases of DLD alone. The combination of comorbid ADHD + DLD (combined or inattentive) did not appear to be associated with lower language abilities, sight word reading, or phonemic decoding. When comparing children and adolescents with comorbid ADHD‐C + DLD to those with comorbid ADHD‐I + DLD, we found that the latter performed worse on nonword repetition tasks. This finding suggests that the presence of a comorbid language disorder primarily affects nonword repetition abilities in children with the inattentive subtype of ADHD. Both combined and inattentive subtypes experience difficulties with attention. Compared to children with hyperactive‐impulsive symptoms, children with inattentive symptoms also experience cognitive control and processing speed weaknesses (Goth‐Owens et al., 2010). These elevated symptoms may have a differential impact on performance in tasks requiring high attentional demands, such as nonword repetition. The subtype of ADHD (combined or inattentive) did not show differential associations with other abilities, except for sight word reading. However, individuals with ADHD‐I still outperformed both comorbid groups on this assessment. A follow up analysis repeated the above ANOVAs excluding test dependent outliers (*n* = 6), and all significant effects remained consistent.

### Diagnostic utility

ROC curves for the four assessments show that they were not all above the reference line or close to the edge of the upper‐left quadrant. Further, the position of the curves changed depending on the discrimination (e.g., ADHD‐C vs. DLD). These findings indicate that no assessments had excellent levels of diagnostic accuracy, and some assessments performed less well with certain discriminations. As expected, the CELFST was very good at distinguishing between the groups that did versus did not have DLD. However, it was not useful at differentiating ADHD‐C from ADHD‐I, or DLD from ADHD‐C + DLD or ADHD‐I + DLD (all AUCs <0.5). The CTOPP nonword repetition was sufficient at distinguishing the groups with versus without DLD, bad at differentiating ADHD‐C from ADHD‐I, and not useful as differentiating DLD from ADHD + DLD. The TOWRE SWE was good at distinguishing ADHD‐C from DLD, ADHD‐C + DLD, and ADHD‐I + DLD, and sufficient at distinguishing ADHD‐I from the groups with DLD. Notably, it was the only measure sufficient in discriminating between the two types of ADHD. It was bad at differentiating DLD from the two groups with ADHD + DLD. The ability of the TOWRE PDE to distinguish between the groups that did versus did not have DLD ranged from good to sufficient. It was bad at discriminating between ADHD‐C and ADHD‐I. Surprisingly, while it was not useful discriminating between DLD and ADHD‐I + DLD, it was sufficient at discriminating DLD from ADHD‐C + DLD.

Overall, the ROC curves demonstrate that within our sample, the CELFST was the best at discriminating most groups of children but particularly those with ADHD from DLD. This finding is expected given that children with ADHD had to pass this screener to be classified as not having comorbid DLD. The other three psycholinguistic measures were good to sufficient at differentiating ADHD from DLD. Most measures were bad to not useful and differentiating between the combined and inattentive subtypes of ADHD but the TOWRE SWE sufficient at doing so. Most measures were also bad to not useful at differentiating DLD from comorbid ADHD + DLD, but the TOWRE PDE did show sufficient ability to discriminating between ADHD‐C + DLD and DLD.

The predictive value of each measure's positive score (or score lower than the cut off) and negative score (or score higher than the cut off) is presented from the positive and negative likelihood ratios in Table 3. The larger the positive likelihood score and smaller the negative likelihood score, the more informative the measure. Under this interpretation, as expected, the CELFST was informative in discriminating between most diagnostic groups that differed in DLD status compared to the remaining assessments which, according to their respective likelihood ratios, were not informative in this regard. Importantly, positive likelihood ratios for all measures, including the CELFST, were not near or above a recommended ratio of 10 (Deeks & Altman, 2004). These findings suggest that test scores on the CELFST below the optimal cut off point for each discrimination are indicative of “positive” rather than very positive of affected language status (Dollaghan, 2007; Redmond, 2011; Sackett et al., 1991). These findings further suggest that lower scores on the CELFST came from participants with DLD, rather than ADHD. On the CELFST, participants' odds of having DLD compared to ADHD‐I increased 5.54 times when they received a raw score below 15. In contrast, the positive likelihood ratio for the CELFST when the discrimination was between ADHD‐C versus DLD was less predictive of DLD status, but still within the “moderately positive” range. The same was found for the CELFST when discriminations were between ADHD‐I and ADHD‐C + DLD. That is, their odds of having ADHD‐C + DLD instead of ADHD‐C or ADHD‐I increased 4.60 and 4.80 times respectively, when they received a raw score below 15. In practical terms, these findings suggest that performance below the cut off scores for the above discriminations are suggestive, but insufficient to assign language disorder status to participants. For negative likelihood ratios, similar findings emerged. All negative ratios associated with informative positive ones on the CELFST were below 0.40, indicating that high scores were “negative” of affected status (Dollaghan, 2007; Redmond, 2011; Sackett et al., 1991). In other words, performance above the cut off values were again suggestive, but not sufficient, to rule out DLD or ADHD‐C + DLD status depending on the discrimination. Together, these findings indicate that, not surprisingly, inadequate performance on the CELFST is suggestive of DLD status but not sufficient to assign a formal language diagnosis. These findings are expected given that the CELFST is a screening test and is not psychometrically set up to diagnose children and adolescents.

**TABLE 3:** Diagnostic Accuracy with psycholinguistic assessments.

Measure	Discrimination	Area under the curve^a^	Optimal cut‐off	Sensitivity	Specificity	Positive likelihood ratio^b^	Negative likelihood ratio^c^
CELF *ST* ^d^	ADHD C versus DLD	0.87	13.00	0.82	0.74	3.15	0.24
	ADHD C versus ADHD C + DLD	0.81	15.00	0.69	0.85	4.60	0.36
	ADHD C versus ADHD I	0.45	12.00	0.91	0.10	1.01	0.90
	ADHD I versus ADHD C + DLD	0.81	15.00	0.72	0.85	4.80	0.33
	ADHD I versus DLD	0.87	15.00	0.72	0.87	5.54	0.32
	ADHD C + DLD versus DLD	0.43	11.00	0.51	0.45	0.93	1.09
	ADHD I versus ADHD I + DLD	0.83	15.00	0.72	0.72	2.57	0.39
	ADHD I + DLD versus DLD	0.47	9.00	0.78	0.30	1.11	0.73
	ADHD C versus ADHD 1 + DLD	0.82	13.00	0.82	0.67	2.48	0.27
CTOPP *NWR* ^e^	ADHD C versus DLD	0.69	7.00	0.66	0.67	2.00	0.51
	ADHD C versus ADHD C + DLD	0.61	5.00	0.86	0.46	1.59	0.30
	ADHD C versus ADHD I	0.51	7.00	0.65	0.35	1.00	1.00
	ADHD I versus ADHD C + DLD	0.60	5.00	0.88	0.46	1.63	0.26
	ADHD I versus DLD	0.68	7.00	0.65	0.67	1.97	0.52
	ADHD C + DLD versus DLD	0.43	5.00	0.53	0.46	0.98	1.02
	ADHD I versus ADHD I + DLD	0.68	7.00	0.65	0.68	2.03	0.51
	ADHD I + DLD versus DLD	0.48	10.00	0.17	0.87	1.31	0.95
	ADHD C versus ADHD I + DLD	0.69	7.00	0.65	0.68	2.03	0.51
TOWRE *SWE* ^f^	ADHD C versus DLD	0.73	100.00	0.75	0.70	2.50	0.36
	ADHD C versus ADHD C + DLD	0.77	96.00	0.79	0.70	2.63	0.30
	ADHD C versus ADHD I	0.62	105.00	0.61	0.55	1.36	0.71
	ADHD I versus ADHD C + DLD	0.68	96.00	0.66	0.71	2.28	0.48
	ADHD I versus DLD	0.63	96.00	0.66	0.60	1.65	0.57
	ADHD C + DLD versus DLD	0.55	94.00	0.50	0.58	1.19	0.86
	ADHD I versus ADHD I + DLD	0.68	97.00	0.63	0.69	2.03	0.54
	ADHD I + DLD versus DLD	0.57	90.00	0.70	0.48	1.35	0.63
	ADHD C versus ADHD I + DLD	0.77	99.00	0.76	0.76	3.17	0.32
TOWRE *PDE* ^f^	ADHD C versus DLD	0.71	93.00	0.76	0.63	2.04	0.38
	ADHD C versus ADHD C + DLD	0.78	97.00	0.69	0.75	2.76	0.41
	ADHD C versus ADHD I	0.57	104.00	0.51	0.55	1.13	0.89
	ADHD I versus ADHD C + DLD	0.76	93.00	0.75	0.67	2.27	0.37
	ADHD I versus DLD	0.69	94.00	0.72	0.67	2.18	0.42
	ADHD C + DLD versus DLD	0.62	85.00	0.77	0.58	1.83	0.40
	ADHD I versus ADHD I + DLD	0.62	96.00	0.67	0.58	1.63	0.56
	ADHD I + DLD versus DLD	0.44	81.00	0.83	0.28	1.15	0.61
	ADHD C versus ADHD I + DLD	0.66	96.00	0.72	0.59	1.76	0.47

Abbreviations: ADHD C, Attention/deficit‐hyperactivity combined type; ADHD C + DLD, Attention‐deficit/hyperactivity disorder and Developmental language disorder; ADHD I, Attention‐deficit/hyperactivity disorder Inattentive type; CELFST, Clinical Evaluation of Language Fundamentals Screening Test; CTOPP, Comprehensive Test of Phonological Processing; DLD, Developmental language disorder; TOWRE, Test of word reading efficiency.

^a^ Optimal cut‐off based on IU, where sensitivity and specificity are the closest to the area under the ROC curve and the absolute value between sensitivity and specificity is minimal.

^b^ Positive likelihood ratio = Sensitivity/(1 − Specificity): Values of 1 = neutral, 3 = moderately positive, ≥10 = very positive.

^c^ Negative likelihood ratio = (1 − Sensitivity)/Specificity: Values of 1 = neutral, ≤0.30 = moderately negative, ≤0.10 = extremely negative.

^d^ Raw Score.

^e^ Scaled Score with *M* = 10, *SD* = 3.

^f^ Standard Score with *M* = 100, *SD* = 10.

In summary, as expected, the CELFST demonstrated high discriminatory ability in distinguishing between ADHD (combined or inattentive) and DLD in children and adolescents. It also performed well in distinguishing between comorbid diagnostic groups. These findings highlight the effectiveness of the CELFST in screening for potential language difficulties. However, additional assessments should be administered following the CELFST to determine specific diagnostic categories and the impact of these categories on language performance. When comparing comorbid cases to “pure” ADHD and DLD, the measures used in the study accurately identified and distinguished DLD and comorbid diagnoses without any decrease in performance. No single task showed superior discrimination between distinct categories, suggesting the importance of utilizing a combination of assessments. Clinical status, particularly DLD or comorbid ADHD‐C + DLD, influenced performance in measures of general language ability, while tasks assessing nonword repetition and phonemic skills were less affected by clinical status. However, it should be noted that inadequate performance on a single assessment was not sufficient to determine clinical status. Further research is needed to validate the findings of the ROC analysis. Overall, these findings highlight the importance of using a comprehensive set of assessments to accurately determine diagnostic categories and understand how clinical status impacts language and reading performance.

### Predicting DLD

In model 1 of the binary logistic regression, nonverbal IQ was entered, and the model was statistically significant χ^2^(1) = 24.40, *p* < .001 but only explained 6% of the variance in diagnosis (Nagelkerske *R* ^2^) and had 68% classification accuracy. Next, the CELFST was entered, and the model was statistically significant, χ^2^(1) = 66.09, *p* < .001, explained an additional 23% of the variance in diagnosis, and improved classification accuracy to 85%. Importantly, once the CELFST was added to the model, nonverbal IQ was no longer a significant predictor of diagnostic status. Adding SWE, PDE, and NWR in the subsequent models did not significantly improve the overall model, classification accuracy, or explain any addition variance (See Table 4 for regression statistics).

**TABLE 4:** Binary regression models for the prediction of developmental language disorder versus all other diagnoses.

Predictors					BCa 95% confidence interval	
	*B*	*SE*	Wald	Exp(*B*)	Lower bound	Upper bound
Model 1						
Nonverbal IQ	−0.046	0.011	3.70*	0.968	0.065	0.026
Model 2						
Nonverbal IQ	−0.019	0.012	0.770	0.985	0.041	0.002
CELFST	−0.264	0.039	16.30**	0.834	0.340	0.189
Model 3						
Nonverbal IQ	−0.015	0.011	0.653	0.985	0.006	0.011
CELFST	−0.237	0.041	15.43**	0.834	0.157	0.041
TOWRE SWE	−0.011	0.014	0.003	0.999	0.017	0.014
TOWRE PDE	−0.004	0.014	1.723	0.979	0.025	0.014
CTOPP NWR	−0.041	0.054	0.024	0.990	0.066	0.055

*Note*: *B* indicates unstandardized regression weights *SE* indicates standard error. Exp(*B*) odds ratio. BCa indicates bias‐corrected accelerated bootstrap interval. Bootstrap confidence intervals are shown for each coefficient. All remaining values are asymptotic.

*p < .05, **p < .001.

These findings indicate that the CELFST was the most efficient measure in predicting DLD status. Given that the CELFST was one of the assessments used to exclude children without potential language difficulties, these findings are not surprising. The cross‐validation analysis indicated that the model was a good fit (RMSE 0.43).

## DISCUSSION

The present study aimed to investigate whether the presence of a comorbid language disorder in ADHD impacts language and reading skills. The study also explored the efficacy of various psycholinguistic assessments in distinguishing between children and adolescents with ADHD, DLD, and comorbid ADHD + DLD. The analyses were guided by prior work examining the diagnostic utility of grammar, nonword repetition, sentence recall, and narrative language measures in ADHD and DLD (Redmond et al., 2011). While language and nonword repetition tasks have been shown to be robust clinical markers of DLD across ages and languages, much less is known about the capacity of reading efficiency measures to serve as clinical markers of DLD. Examining the capabilities of language and reading in distinguishing ADHD, DLD, and comorbid ADHD + DLD could lead to the adoption of these assessments in future protocols and further elucidate the overlapping difficulties experienced by these etiologically distinct disorders.

The current study demonstrated that children with ADHD (combined and inattentive) have similar oral language skills, and reading efficiency measures are capable of distinguishing between subtypes of ADHD. However, despite these strengths, none of the groups could be clearly defined based solely on their reading or language abilities. Additional assessments are therefore necessary to accurately identify the presence of ADHD and/or DLD status.

The findings of the current study indicate that the coexistence of ADHD in children with DLD does not exacerbate language and reading difficulties. Moreover, the significant differences between children and adolescents with ADHD (combined and inattentive) were observed only in SWE, in favor of those with combined type. Cross‐validation methods demonstrated that the model had good generalizability, suggesting that the results can be extended to other samples in predicting DLD status using the combination of tasks employed in the current study. Importantly, while reading efficiency measures, specifically SWE, proved to be the most effective in distinguishing between the two types of ADHD, the CELFST demonstrated the best overall performance in distinguishing between pure and comorbid cases of ADHD + DLD. The CELFST also emerged as the strongest predictor of diagnostic status, surpassing nonverbal IQ.

Our findings diverge from previous studies showing high classification accuracy of DLD on nonword repetition tasks (Redmond et al., 2011). While the CELFST was found to be the best at distinguishing between ADHD and DLD, both reading efficiency subtests (SWE and PDE) performed well and were not far behind. In cases where the CELFST fell short in distinguishing between groups, such as in DLD versus comorbid ADHD‐C + DLD, the SWE and PDE measures demonstrated good discriminatory abilities.

It is important for cut‐off values to be replicated in other settings to determine the true accuracy of diagnostic tests (Redmond et al., 2011; Sackett & Haynes, 2002). As mentioned by Redmond (2011), optimal cut‐off values can vary across studies and may be based on arbitrary criteria, such as “1.0 *SD* below the mean or below the 10^th^ percentile”. Moreover, certain cut‐off scores are more advantageous than others due to their standardized nature. Notably, all assessments in the current study except for the CELFST provide valuable cut‐off information as they are presented as standard or scaled scores, ensuring normalization across various age groups. However, because the CELFST relies on criterion referencing, the optimal cut‐off point of 15 identified in the current study would not be applicable to any individual child. It is crucial to emphasize that the current study primarily focused on understanding issues around comorbidity and symptom severity rather than clinical diagnoses based solely on these assessments. Thus, discussions regarding the alignment between cut‐off scores and expectations based on clinical norms should be approached with caution.

Variability in estimates of comorbidity between ADHD and DLD symptoms can be attributed to differences in study design elements, such as age range, inclusionary and exclusionary criteria, and diagnostic criteria. Reports of co‐occurrence between ADHD and DLD can range widely, from 8% to 90% in adults (Tannock & Schachar, 1996) and 4% to 35% in children (Cantwell & Baker, 1987; Snowling et al., 2006). These variations highlight the importance of using reliable assessments in studying comorbidity. Currently, there is a lack of sufficient investigations comparing pure and comorbid samples of ADHD and DLD. The current study is the first to evaluate the diagnostic integrity of language and reading measures in children and adolescents who meet the criteria for both language impairment and ADHD. These investigations can assist researchers in interpreting co‐occurring symptoms and determining which assessments can serve as valid clinical markers in similar groups of children. Although the CELFST alone is not sufficient to assign a diagnosis of language impairment, it contains some of the most discriminating items from the full Clinical Evaluation of Language Fundamentals— Screening Test, which is frequently used as a benchmark or reference standard in language impairment assessments.

Contrary to previous research (Conti‐Ramsden, 2003; Conti‐Ramsden & Hesketh, 2003; Oetting & Cleveland, 2006; Poll et al., 2010; Redmond et al., 2011), the nonword repetition task used in the current study did not accurately distinguish between any of the groups. The discrepancy in findings could be attributed to several factors, particularly differences in task designs across studies. The most used nonword repetition task is an adaptation of Dollaghan and Campbell's (1998) task, known as the NWR. This task has been shown to distinguish children with DLD from their TD peers (Graf et al., 2007) and more recently, children with ADHD from those with DLD (Redmond et al., 2011). However, when combined with additional language assessments, the NWR task has demonstrated high diagnostic accuracy but low sensitivity rates that limit its clinical utility (50% range; Conti‐Ramsden, 2003; Oetting & Cleveland, 2006). Another nonword repetition task used in younger children is the Children's Test of Nonword Repetition (CNRep; Gathercole & Baddeley, 1996). It shares similar features and scoring procedures with Dollaghan and Campbell's NWR task but presents 40 nonwords of varying syllable lengths. In the current study, the CTOPP‐2 was used, which is almost identical to the previous assessments but includes 30 nonwords increasing in complexity. The CTOPP‐2 has shown higher construct validity on both subtest and composite scores compared to the CNRep (Tennant, 2014). When paired with valid language assessments, the CTOPP‐2 is considered a robust and reliable tool for identifying DLD in numerous studies (Leyfer et al., 2008; Loucas et al., 2016; Paradis, 2016). There are also differences in the way NWR tasks can be scored, which can impact their ability to distinguish groups (Archibald & Joanisse, 2009). For example, some researchers score these tasks by deducting points for phonemic errors using offline transcriptions. Other researchers score assessments online where correct responses depend on accurate recall at the item‐level. Item‐level scoring is argued to be more clinically practical because it minimizes training needs. The NWR used in the current study was scored at the item‐level. Despite these task differences, the findings of the current study aligned with previous research, showing that children with DLD produced significantly more phonological errors compared to those with ADHD. These findings suggest that the deficits observed in DLD may not be dependent on the specific nonword repetition task used or the way it is scored.

The most interesting finding of the current study was that reading efficiency, rather than oral language, was the best discriminator between ADHD subtypes. The results from the group difference analyses supported this, revealing significant differences between children and adolescents with ADHD‐I and ADHD‐C in their ability to identify real words (TOWRE SWE), in favor of those with ADHD‐C. However, there were no significant differences between the two groups in their ability to identify nonwords (TOWRE PDE). Inattention symptoms have been found to have a greater impact on sight word reading compared to hyperactive‐impulsive symptoms (Martinussen et al., 2014). Nonetheless, children with ADHD‐C also exhibit deficits in attention. An alternative, but related explanation is that children and adolescents with ADHD‐C may benefit from the speeded nature of the SWE subtest due to their more impulsive style. Children with the predominately inattentive subtype also have greater processing speed weaknesses than children with hyperactive‐impulsive symptoms (Goth‐Owens et al., 2010) which may result in difficulties recalling words quickly. Children with the inattentive subtype may possess the knowledge of words but may not be as quick at identifying them during the task.

A more plausible explanation is that the ADHD‐I group has higher levels of reading difficulties that do not reach clinical significance compared to the ADHD‐C group. The current study excluded children with comorbid ADHD + reading disorders. Previous research has shown that inattention symptoms predict later reading achievement, even after controlling for core reading skills, hyperactivity, and reading levels (Miller et al., 2014; Rabiner & Coie, 2000). The findings of the current study suggest that inattention, even in the absence of a specific reading disorder, poses a risk to reading difficulties. These results highlight the importance of assessing whether children with ADHD exhibit reduced sight word and decoding skills, which are crucial for successful reading. They also underscore the important connections between impulsivity, attention, and reading.

To distinguish ADHD subtypes, performance on the SWE subtest of the TOWRE could be useful when combined with other well‐validated assessments. Additionally, children and adolescents with ADHD‐I may benefit from treatments focused on improving sight word reading. However, further research is needed to replicate these findings and enhance our understanding of ADHD subtypes and their relationship to reading efficiency.

The pattern of findings from the present study have several important implications. First, the presence of an additional diagnosis of ADHD in children with DLD does not compound language and reading difficulties further. Second, as expected, the CELFST distinguished children with DLD from those with ADHD, including most comorbid cases. However, there are exceptions when it comes to distinguishing between ADHD subtypes and comorbid ADHD‐C + DLD from DLD. In these cases, the reading efficiency subtests show better performance. Therefore, the findings suggest that when distinguishing between ADHD and DLD, the use of both assessments (CELFST and TOWRE) will lead to more accurate outcomes. Additionally, it is important to consider individual challenges in language and reading within each diagnostic group, even when assessments can reliably distinguish between the groups.

## CONCLUSIONS AND FUTURE DIRECTIONS

Our findings have implications for both clinicians and researchers in terms of differential diagnosis and the identification of comorbidity. The results indicated that assessments of reading efficiency may be useful in distinguishing between different subtypes of ADHD. However, none of the groups could be clearly defined based solely on their reading or language abilities, highlighting the need for additional assessments to identify ADHD and/or DLD status.

Future research should focus on investigating which assessments are most effective in distinguishing between comorbid groups and different subtypes of ADHD and DLD, as well as other related disorders. The current findings suggest that assessments of reading efficiency may be a productive starting point. Furthermore, it is important to explore whether the same set of assessments can accurately predict DLD and/or ADHD in both younger and older children. While nonword repetition tasks have been established as robust markers of language impairment in young adults (Poll et al., 2010) and children (Conti‐Ramsden, 2003; Conti‐Ramsden & Hesketh, 2003; Oetting & Cleveland, 2006; Redmond et al., 2011), their applicability to ADHD and comorbid samples remains unclear and warrants further investigation.

In addition to behavioral assessments, future research should also explore the potential of physiological markers, like electroencephalography (EEG), in distinguishing between ADHD, DLD, and comorbid samples. Studies have shown atypical EEG patterns in children with ADHD (Barry et al., 2003, 2010; Koehler et al., 2009; Satterfield et al., 1974), and specific EEG patterns have been approved by the Food and Drug Administration for informing ADHD diagnosis. There are also connections between specific EEG patterns and language proficiency (Beese et al., 2017; Hald et al., 2006; Lume et al., 2022). Therefore, further research is needed to explore the potential of objective physiological metrics in distinguishing between ADHD and comorbid ADHD + DLD. Investigating the utility of such metrics can provide valuable insights into the underlying biological markers and help improve the diagnostic accuracy of these conditions.

The current study has certain limitations that should be acknowledged. The sample used in the study did not include individuals with the hyperactive‐impulsive subtype of ADHD, limiting the generalizability of the findings to the broader population of individuals with ADHD. Moreover, the high heterogeneity of the disorders examined and the small sample sizes in some comorbid subgroups may have reduced statistical power and generalizability. Future studies should aim to include larger samples to enhance the confidence in the reported findings. The current study did not include a non‐clinical comparison group because the primary aim was to assess whether having both conditions worsened difficulties and contribute to the lack of studies performing cross‐clinical comparisons. It is essential for future research to explore whether these findings can extend to other diagnostic groups with related difficulties. Given the frequent co‐occurrence of ADHD, DLD, and reading disorders, it is crucial to examine whether the assessments used in the current study can effectively distinguish these populations. Further research is warranted to explore these aspects and broaden our understanding of these disorders.

## AUTHOR CONTRIBUTIONS

**Kaitlyn M. A. Parks**: Conceptualization; formal analysis; investigation; methodology; project administration; writing – original draft; writing – review & editing. **Janis Oram Cardy**: Writing – review & editing. **Marc F. Joanisse**: Resources; software; supervision; validation; visualization; writing – review & editing.

## CONFLICT OF INTEREST STATEMENT

The authors have declared that they have no competing or potential conflicts of interest.

## ETHICAL CONSIDERATIONS

The original HBN study was approved by the Chesapeake Institutional Review Board (https://www.Chesapeakeirb.com/). Prior to conducting the research, written informed consent was obtained from participants ages 18 years or older. For participants younger than 18, written consent was obtained from their legal guardians and written assent obtained from the participant.

## Supporting information

Supporting Information S1

## Data Availability

The data that support the findings of this study are available in the Healthy Brain Network Data Portal at [https://fcon_1000.projects.nitrc.org/indi/cmi_healthy_brain_network/Data%20Quality.html].
