# Supplementary material for: Language and reading in attention‐deficit/hyperactivity disorder and comorbid attention‐deficit/hyperactivity disorder + developmental language disorder
Source: JCPP Adv. 2024 Apr 17;4(2):e12218. doi: 10.1002/jcv2.12218 (PMC11143959; doi:10.1002/jcv2.12218)
Supplement: Supplementary file 1 — Supporting Information S1 [file JCV2-4-e12218-s001.docx]

Supporting Materials


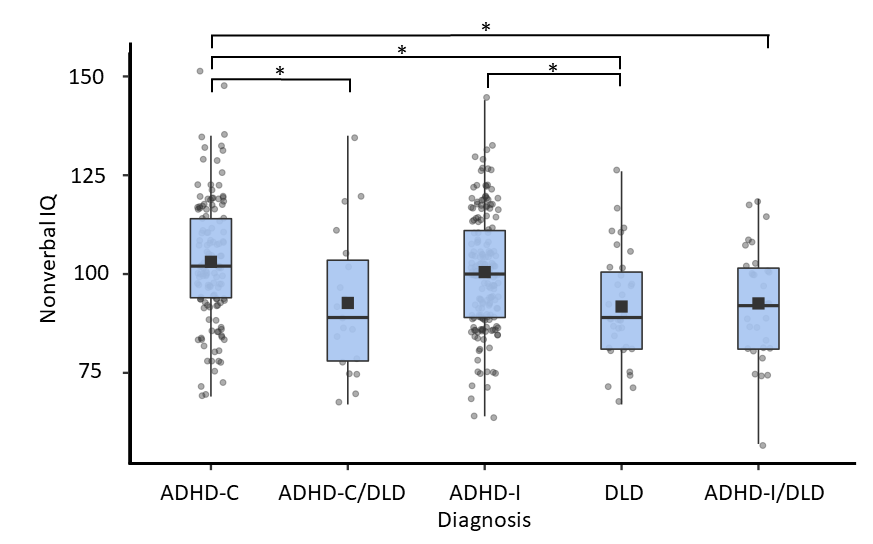


**Figure S1.** Box plots for Visual Spatial Index (VSI scores from The Wechsler Intelligence Scale (WISC-V). ADHD C = Attention-deficit/hyperactivity disorder combined type; ADHD I = ADHD inattentive type; DLD = Developmental language disorder.


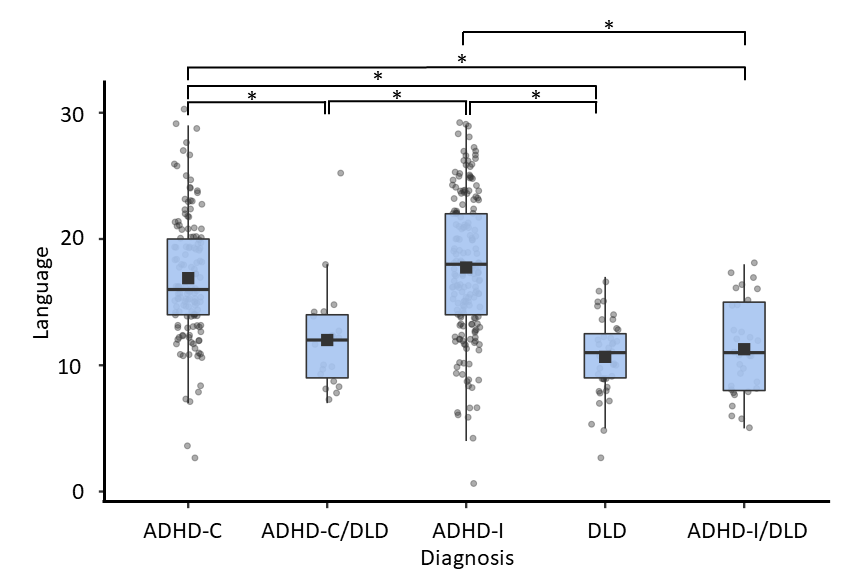


**Figure S2.** Box plots for Clinical Evaluation of Language Fundamentals – Screening Test (CELFST). ADHD C = Attention-deficit/hyperactivity disorder combined type; ADHD I = ADHD inattentive type; DLD = Developmental language disorder.


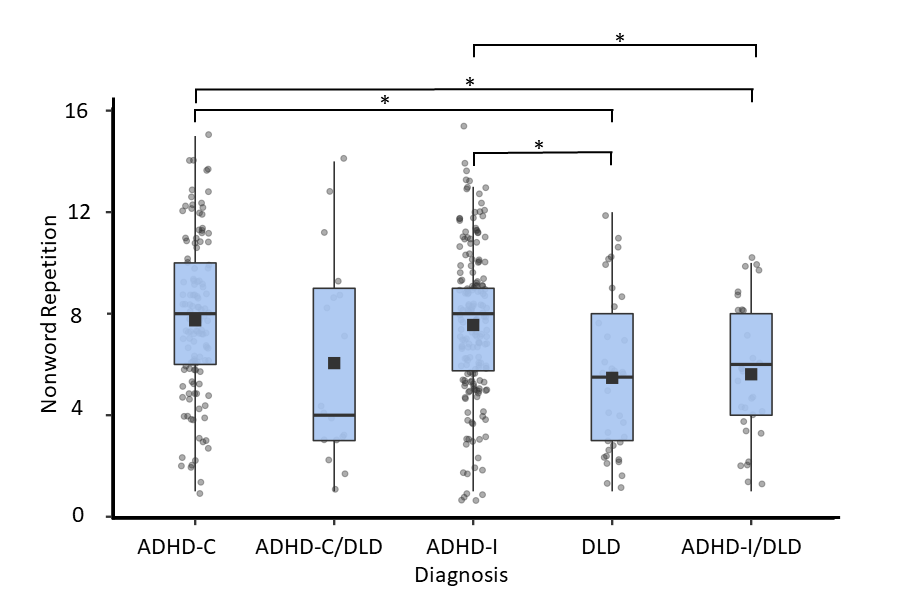


**Figure S3.** Box plots for Comprehensive Test of Phonological Processing (CTOPP) Nonword Repetition (NWR) subscale scores. ADHD C = Attention-deficit/hyperactivity disorder combined type; ADHD I = ADHD inattentive type; DLD = Developmental language disorder.


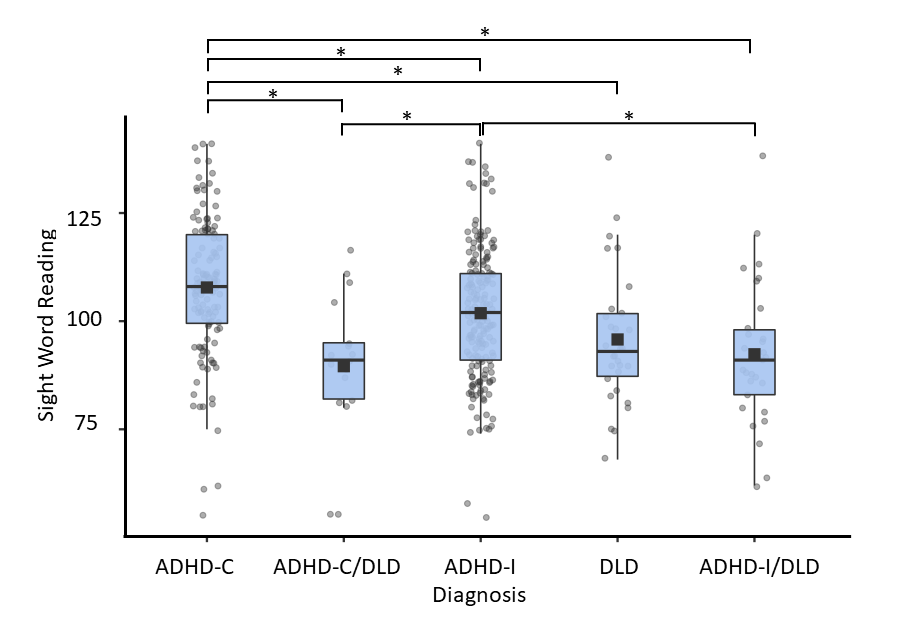


**Figure S4.**  Box plots for Sight Word Efficiency (SWE) reading scores. ADHD C = Attention-deficit/hyperactivity disorder combined type; ADHD I = ADHD inattentive type; DLD = Developmental language disorder.


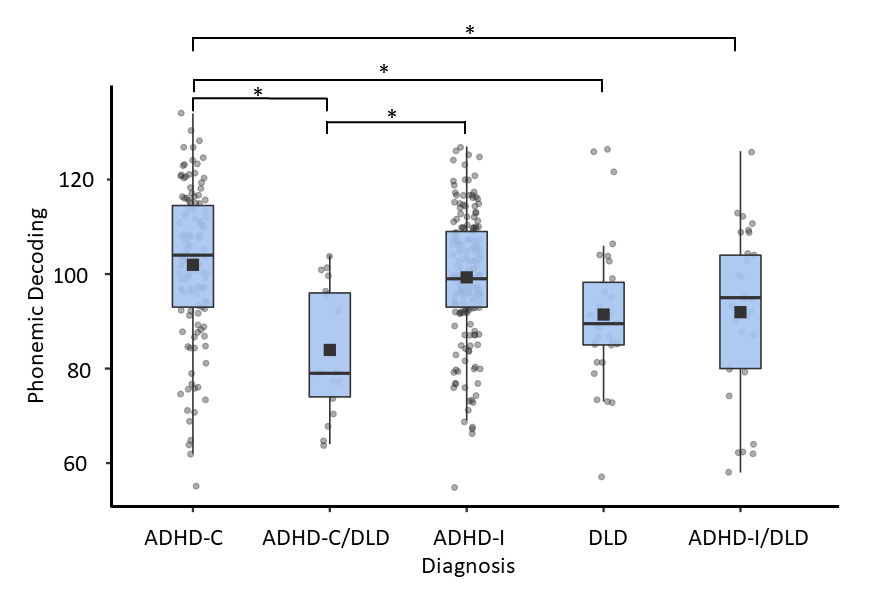


**Figure S5.** Box plots for Phonemic Decoding Efficiency (PDE) reading scores. ADHD C = Attention-deficit/hyperactivity disorder combined type; ADHD I = ADHD inattentive type; DLD = Developmental language disorder.

ADHD Combined versus ADHD Combined + DLD

| **CELFST** **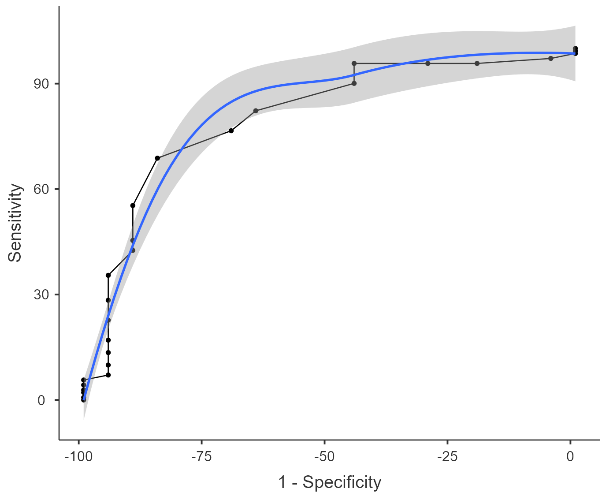** | **CTOPP_NWR** **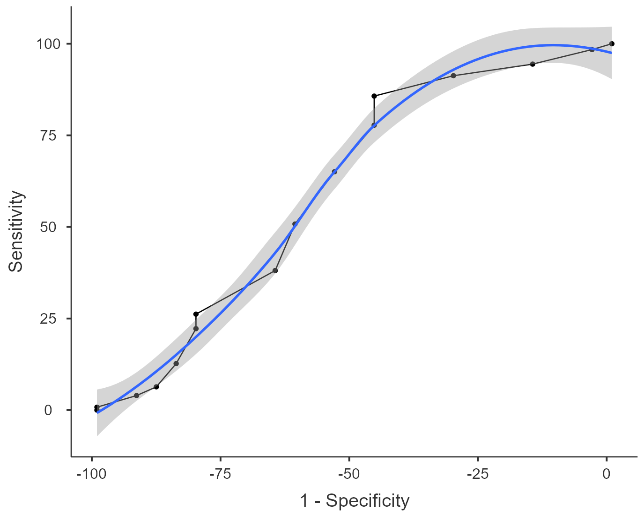** |
| --- | --- |
| **TOWRE_SWE** **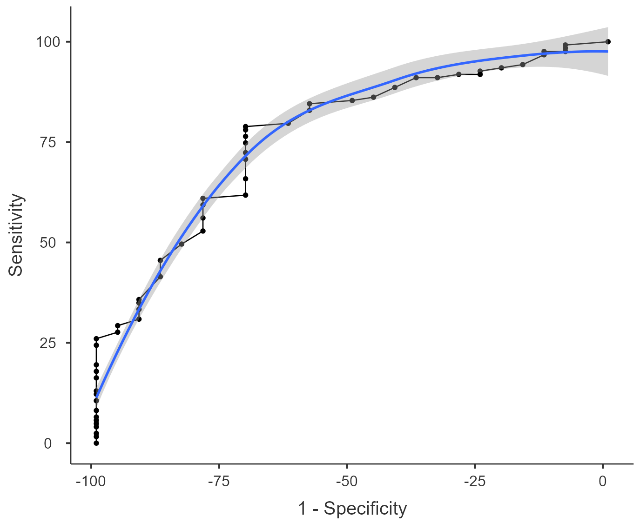** | **TOWRE_PDE** **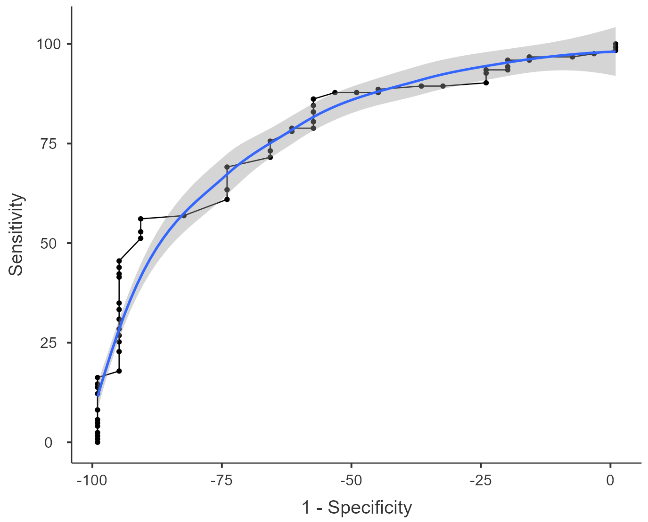** |

**Figure S6.**

Receiver operating characteristics (ROC) curves associated with linguistic discrimination of ADHD combined versus ADHD combined + DLD. 95 CI bound and standard error bars included (grey reference line indicates test accuracy at “chance”).

ADHD Combined versus DLD

| **CELFST**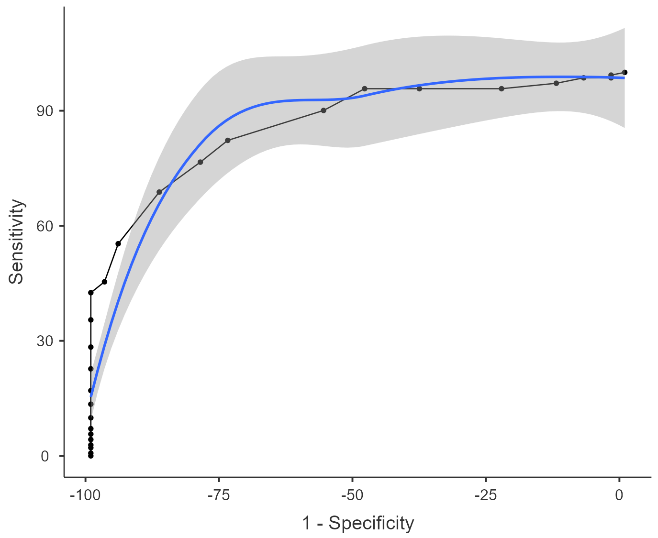 | **CTOPP_NWR** 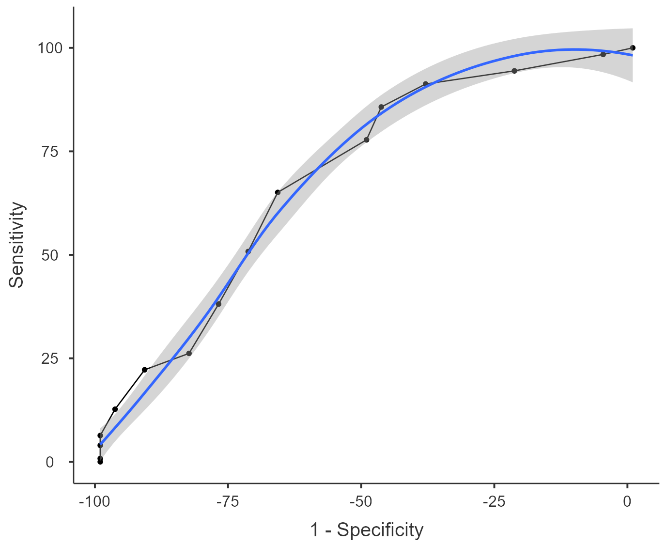 |
| --- | --- |
| **TOWRE_SWE** 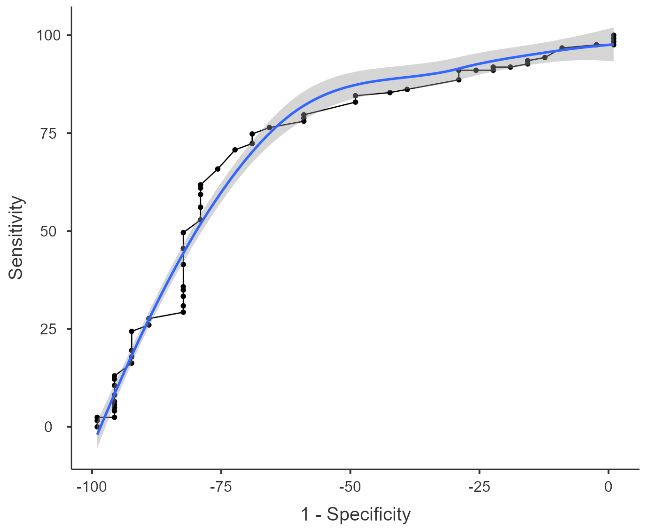 | **TOWRE_PDE** 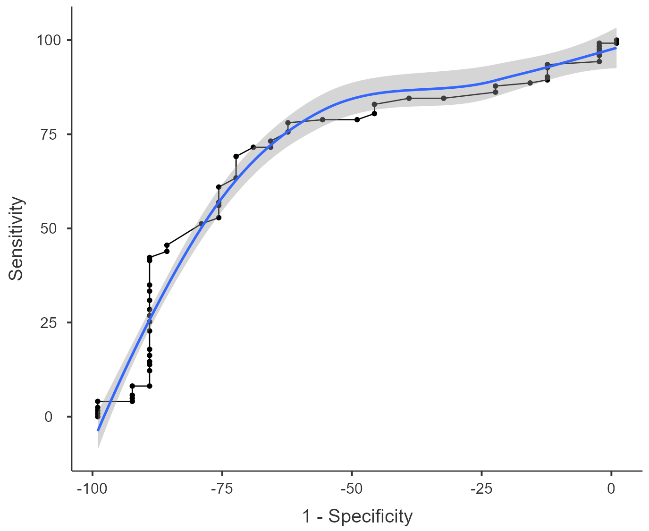 |

**Figure S7.**

Receiver operating characteristics (ROC) curves associated with linguistic discrimination of ADHD combined versus DLD groups. 95 CI bound and standard error bars included (grey reference line indicates test accuracy at “chance”).

ADHD Inattentive versus DLD

| **CELFST** 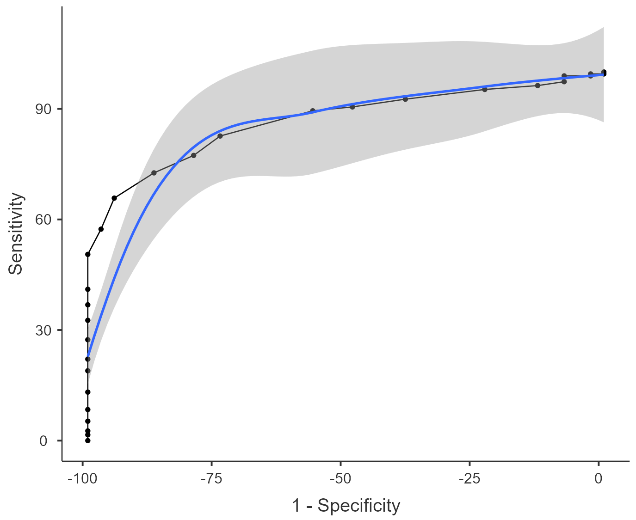 | **CTOPP_NWR** 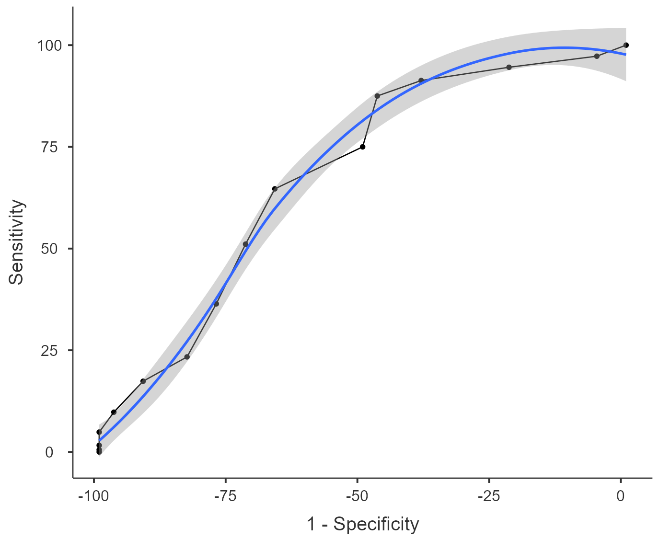 |
| --- | --- |
| **TOWRE_SWE** 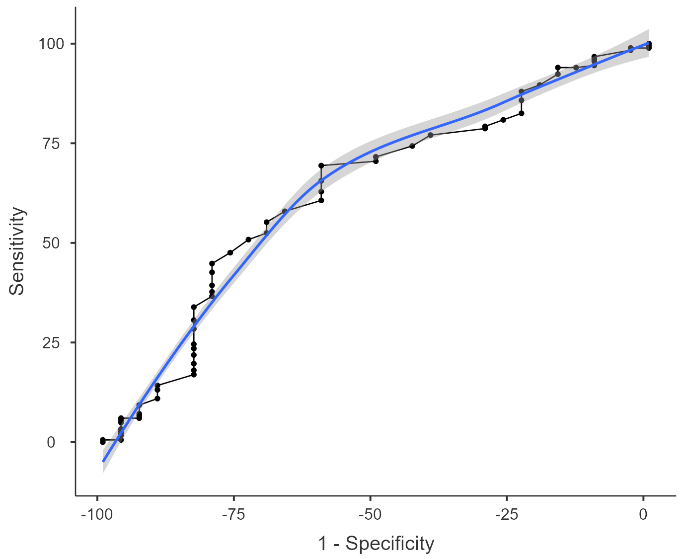 | **TOWRE_PDE** 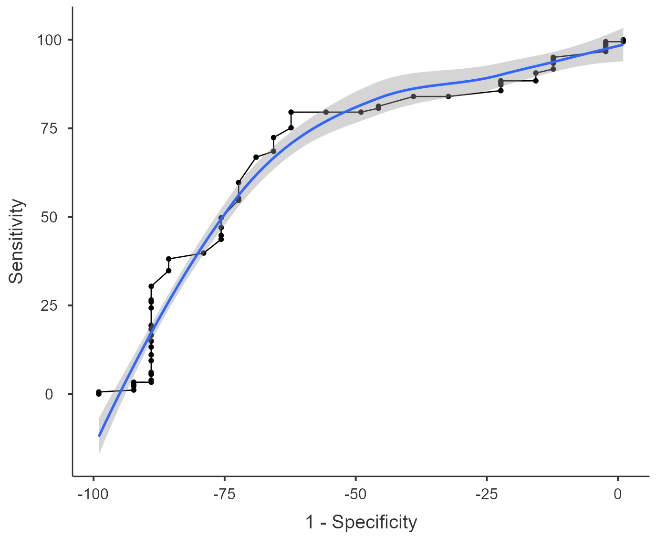 |

**Figure S8.**

Receiver operating characteristics (ROC) curves associated with linguistic discrimination of ADHD inattentive versus DLD groups. 95 CI bound and standard error bars included (grey reference line indicates test accuracy at “chance”).

ADHD Combined + DLD versus DLD

| **CELFST** 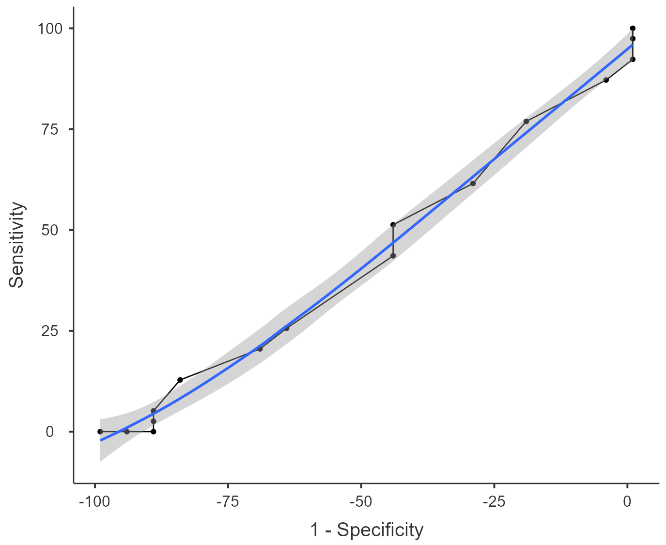 | **CTOPP_NWR** 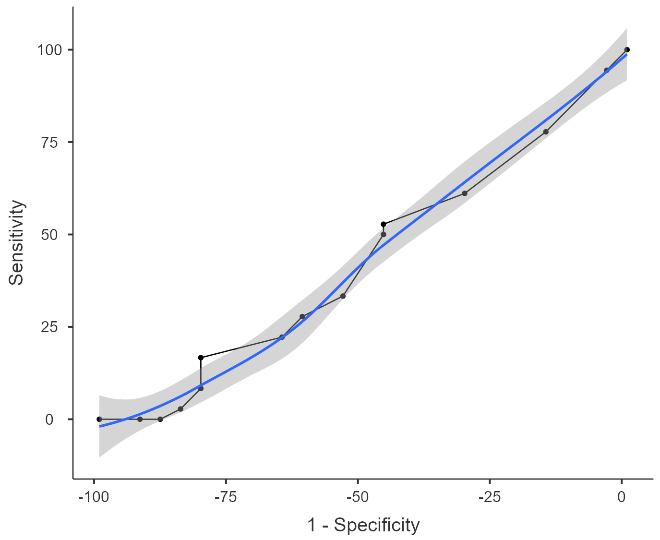 |
| --- | --- |
| **TOWRE_SWE** 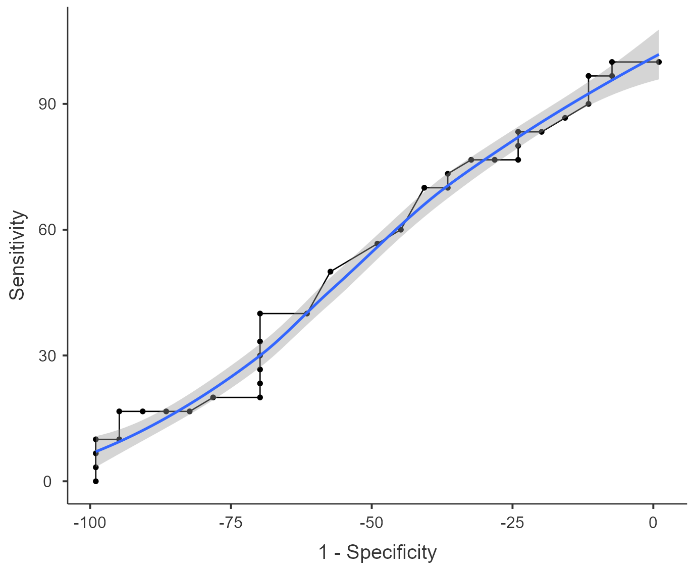 | **TOWRE_PDE** 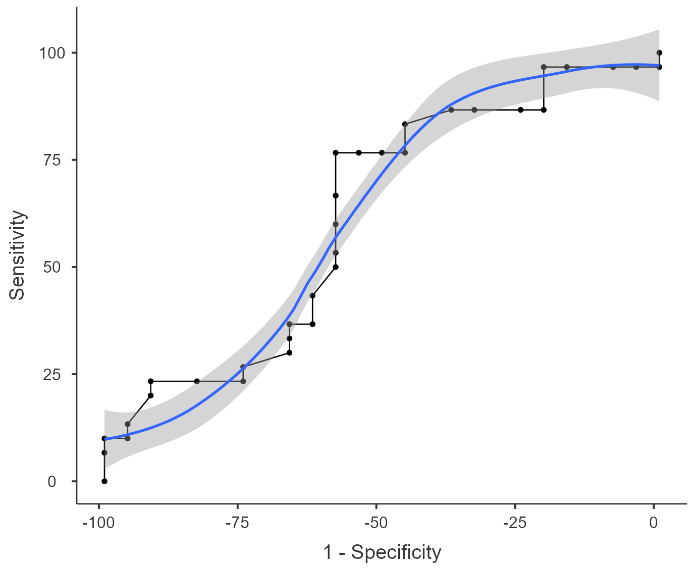 |

**Figure S9.**

Receiver operating characteristics (ROC) curves associated with linguistic discrimination of ADHD combined + DLD versus DLD groups. 95 CI bound and standard error bars included (grey reference line indicates test accuracy at “chance”).

ADHD Combined + DLD versus ADHD Inattentive

| **CELFST** 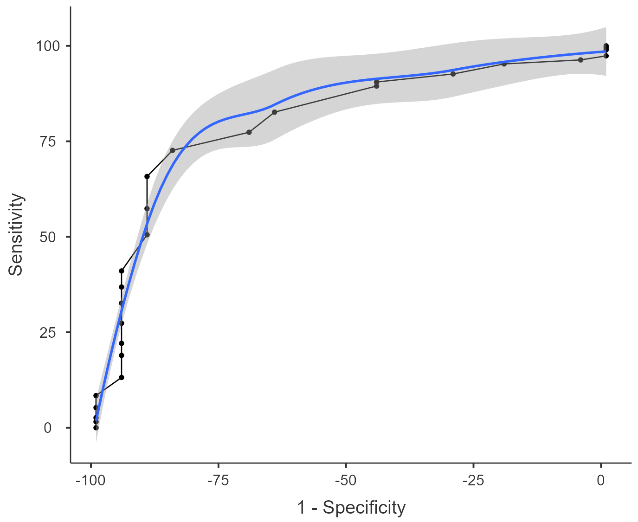 | **CTOPP_NWR** 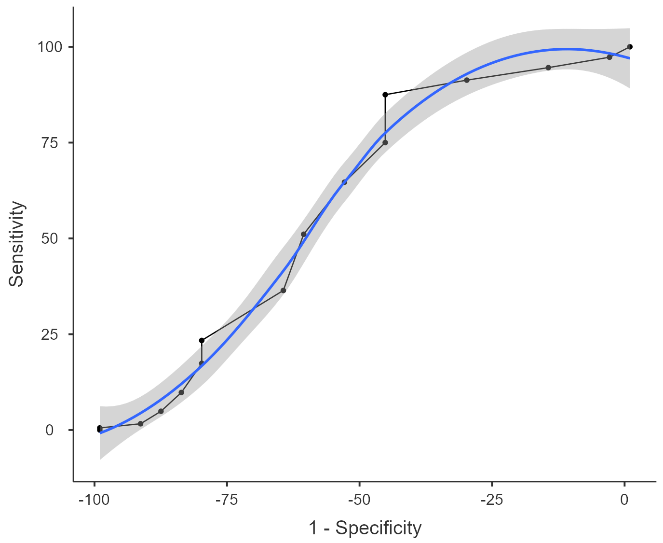 |
| --- | --- |
| **TOWRE_SWE** 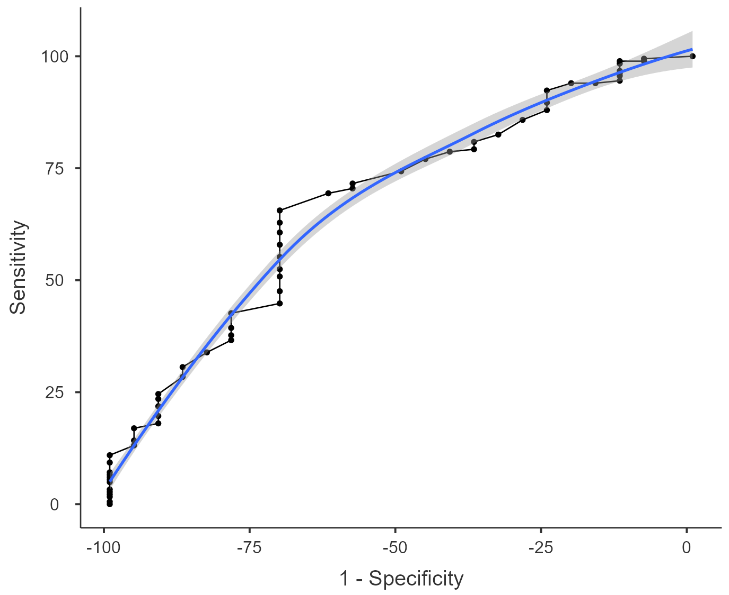 | **TOWRE_PDE** 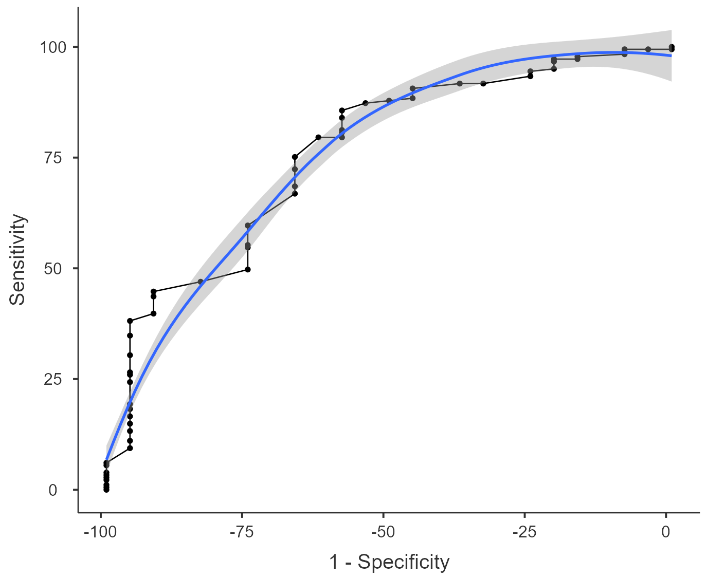 |

**Figure S10.**

Receiver operating characteristics (ROC) curves associated with linguistic discrimination of ADHD combined + DLD versus ADHD inattentive groups. 95 CI bound and standard error bars included (grey reference line indicates test accuracy at “chance”).

ADHD Combined versus ADHD Inattentive

| **CELFST** 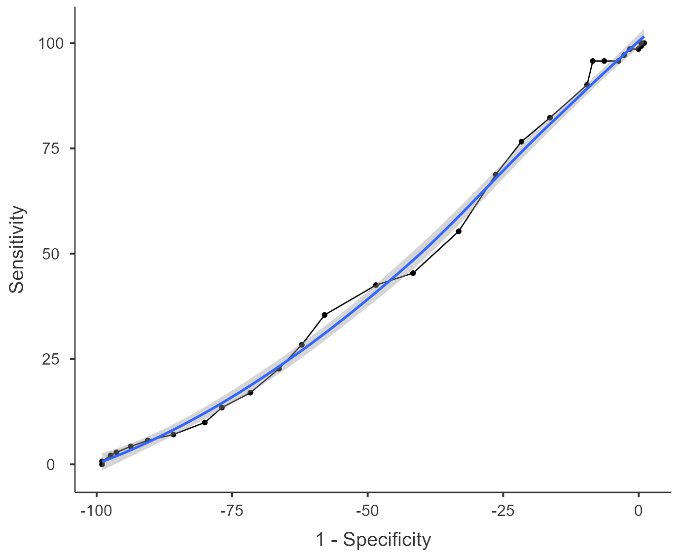 | **CTOPP_NWR** 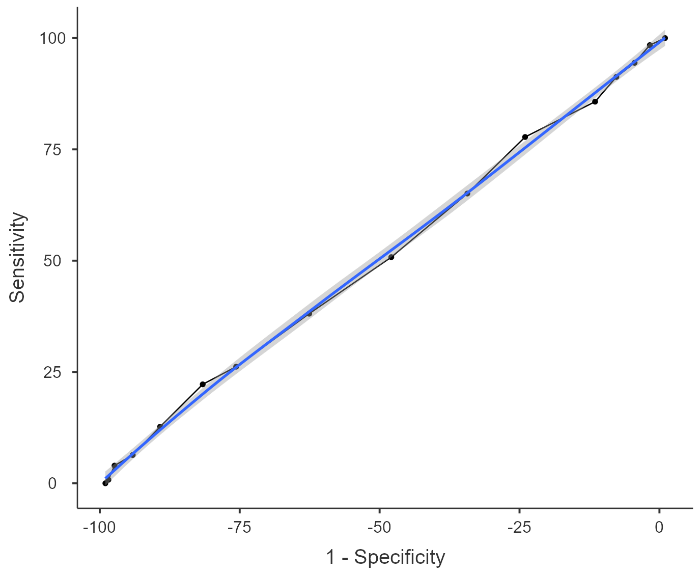 |
| --- | --- |
| **TOWRE_SWE** 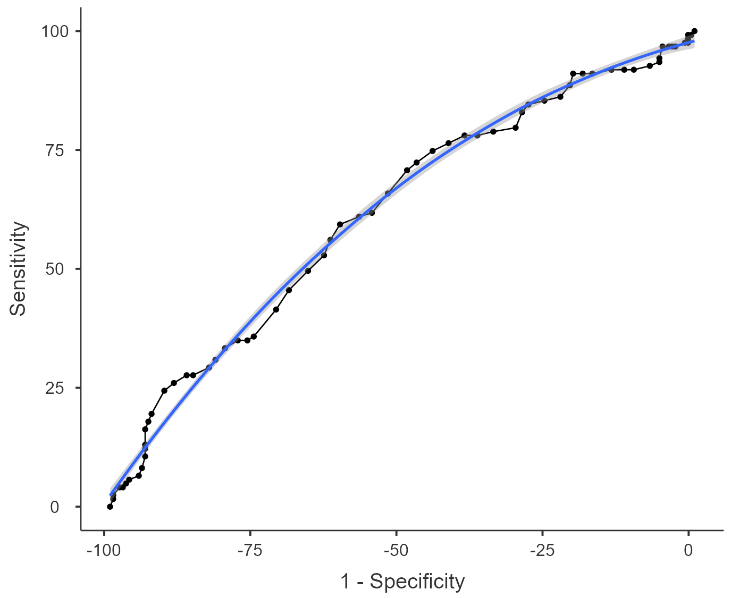 | **TOWRE_PDE** 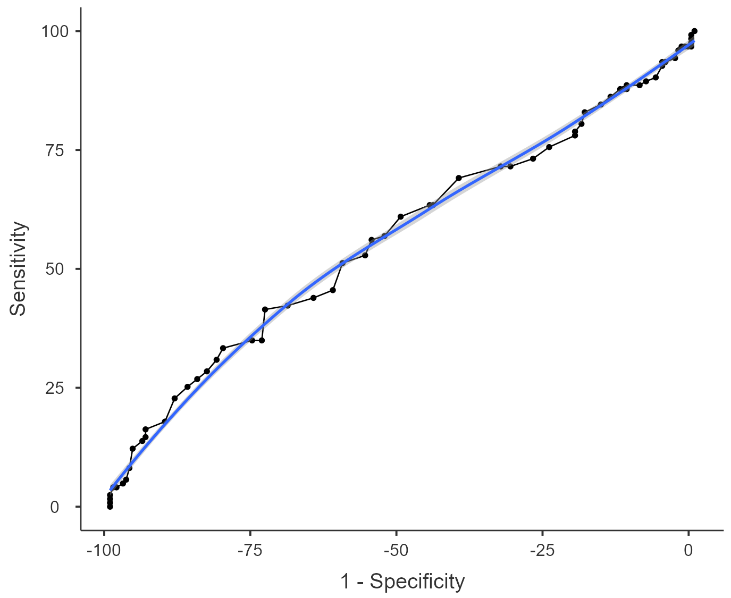 |

**Figure S11.**

Receiver operating characteristics (ROC) curves associated with linguistic discrimination of ADHD combined versus ADHD inattentive groups. 95 CI bound and standard error bars included (grey reference line indicates test accuracy at “chance”).

ADHD Combined versus ADHD Inattentive + DLD

| **CELFST** 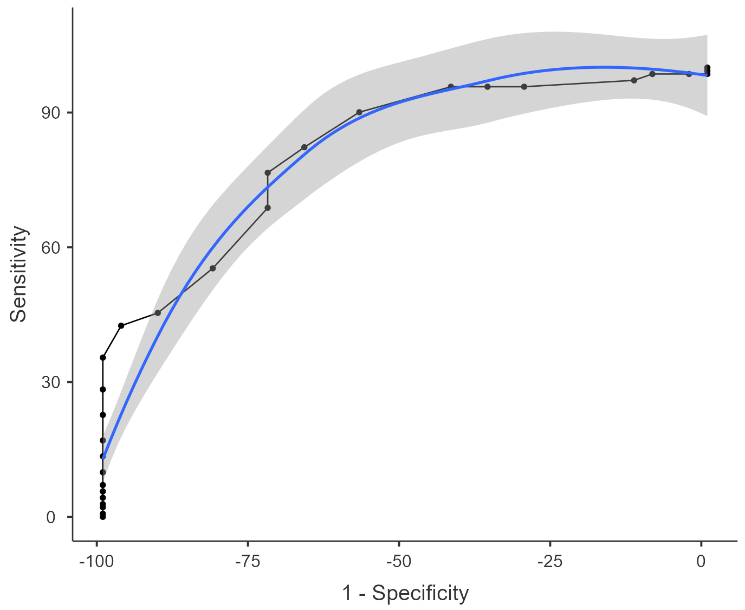 | **CTOPP_NWR** 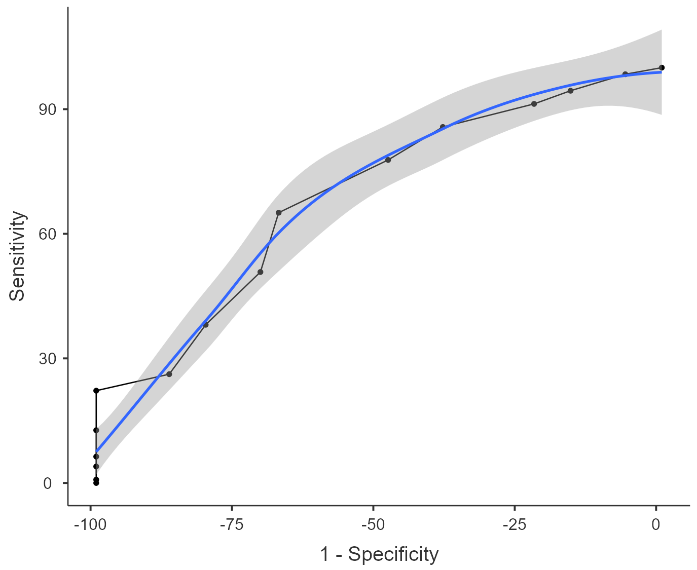 |
| --- | --- |
| **TOWRE_SWE** 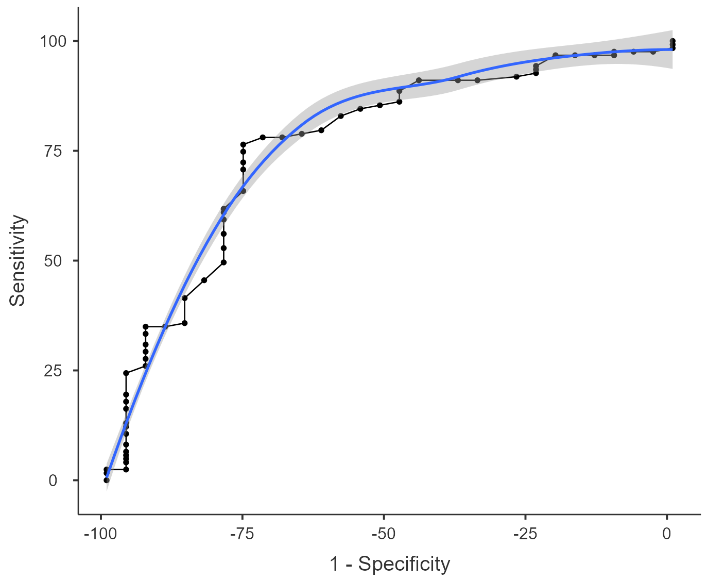 | **TOWRE_PDE** 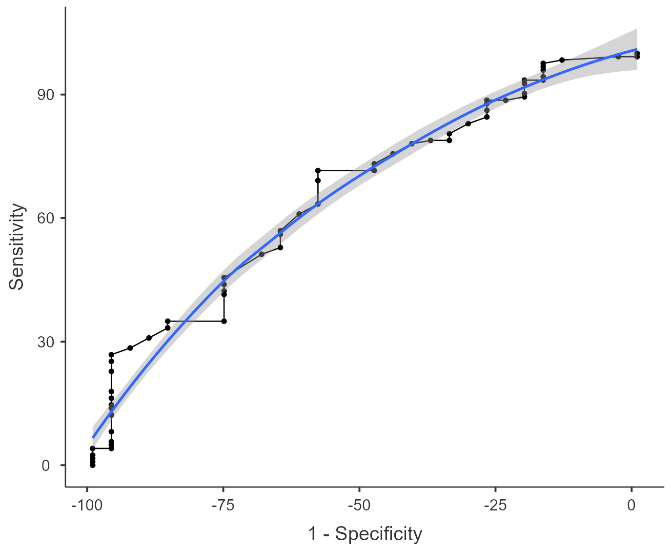 |

**Figure S12.**

Receiver operating characteristics (ROC) curves associated with linguistic discrimination of ADHD combined versus ADHD inattentive + DLD groups. 95 CI bound and standard error bars included (grey reference line indicates test accuracy at “chance”).

ADHD Inattentive versus ADHD Inattentive + DLD

| **CELFST** 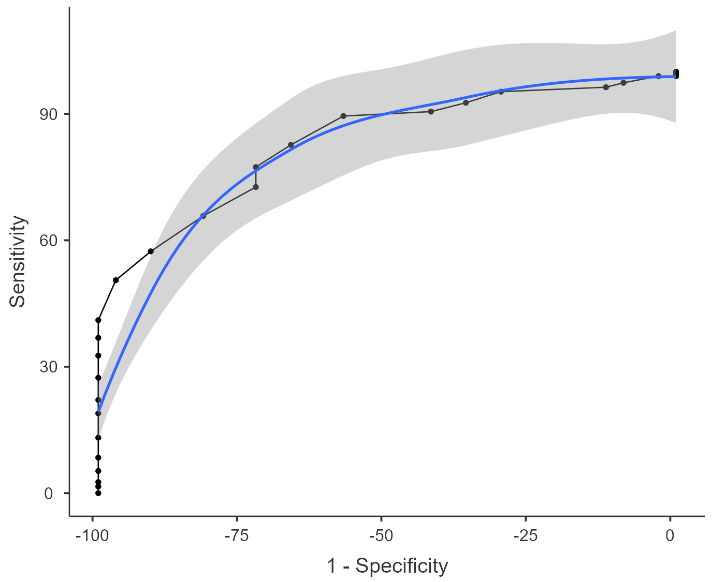 | **CTOPP_NWR** 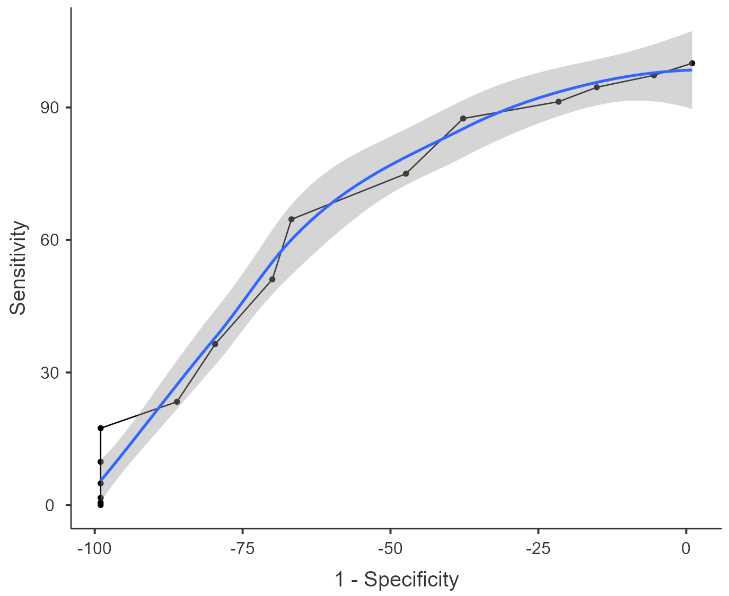 |
| --- | --- |
| **TOWRE_SWE** 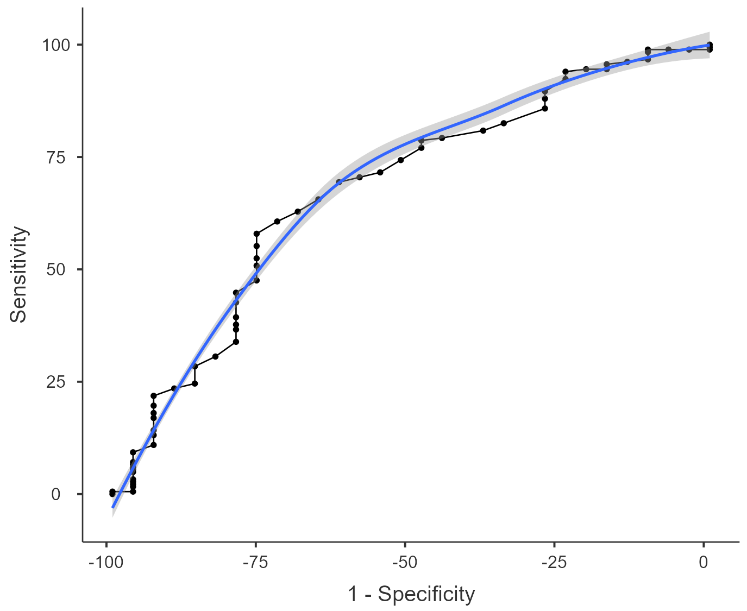 | **TOWRE_PDE** 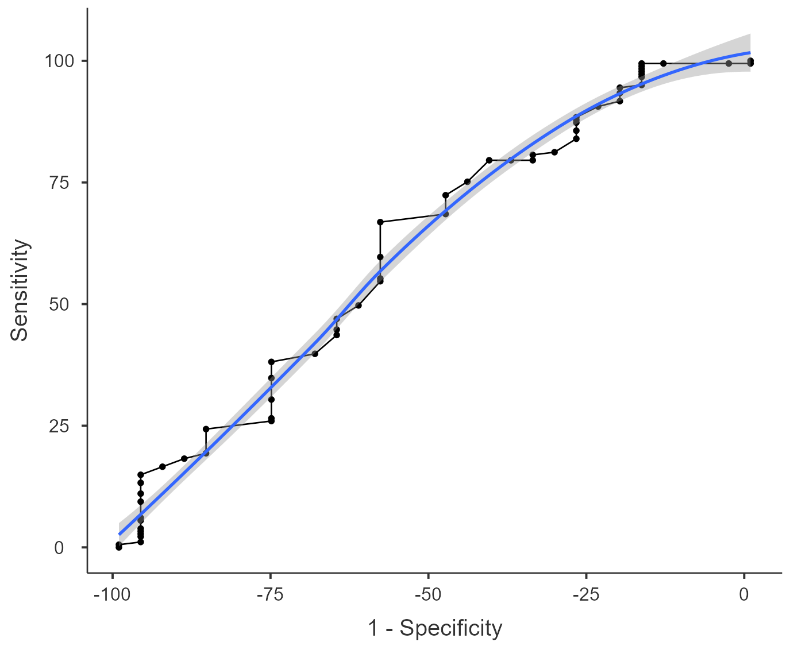 |

**Figure S13.**

Receiver operating characteristics (ROC) curves associated with linguistic discrimination of ADHD inattentive versus ADHD inattentive + DLD groups. 95 CI bound and standard error bars included (grey reference line indicates test accuracy at “chance”).

ADHD Inattentive + DLD versus DLD

| **CELFST** 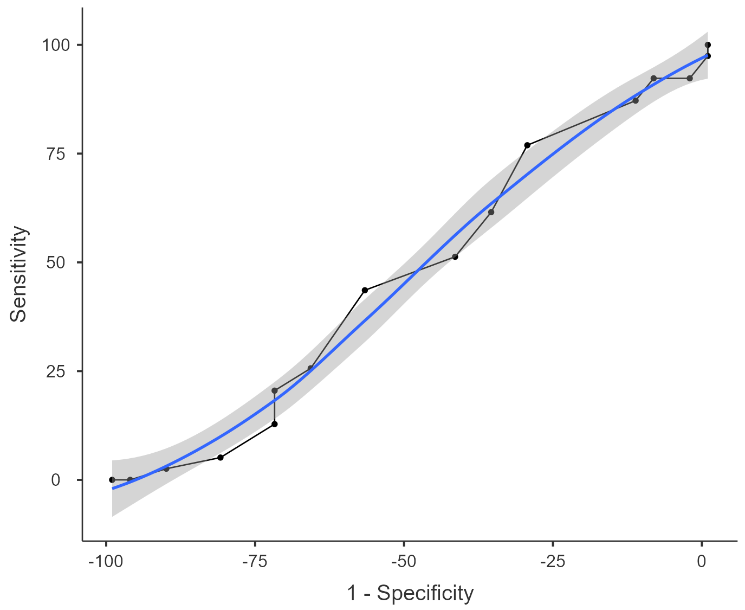 | **CTOPP_NWR** 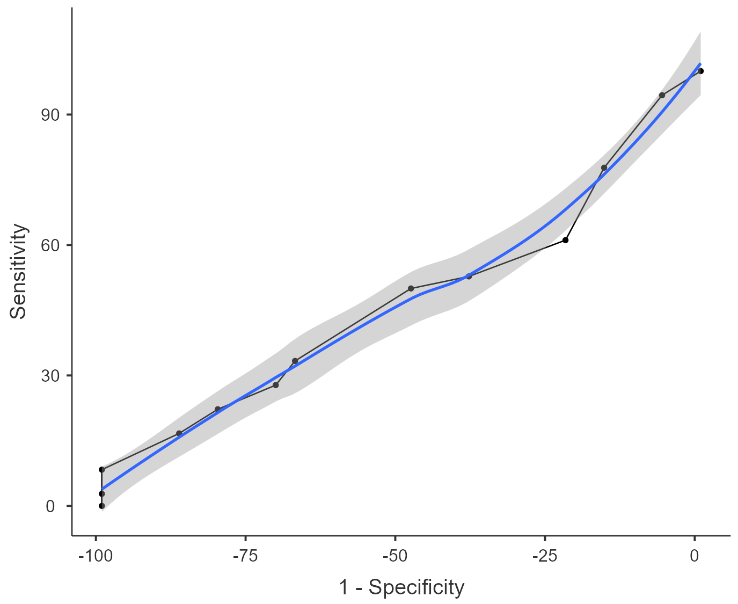 |
| --- | --- |
| **TOWRE_SWE** 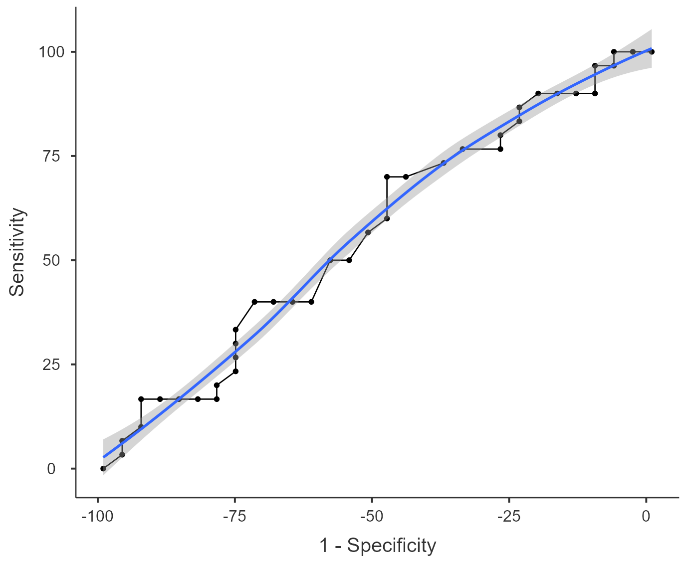 | **TOWRE_PDE** 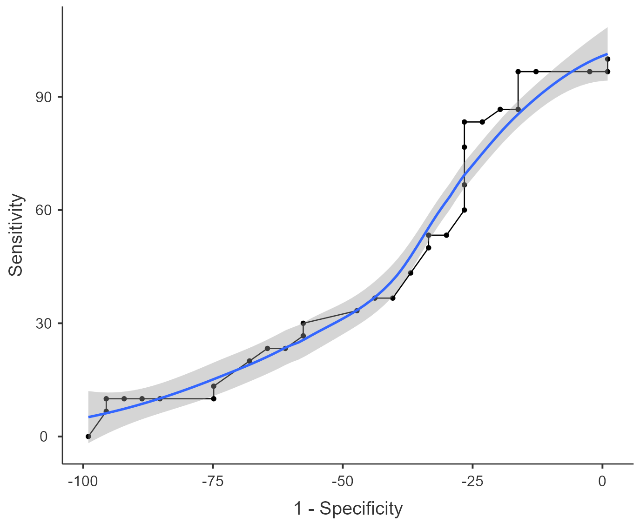 |

**Figure S14.**

Receiver operating characteristics (ROC) curves associated with linguistic discrimination of ADHD inattentive + DLD versus DLD groups. 95 CI bound and standard error bars included (grey reference line indicates test accuracy at “chance”).
